# SARS-CoV-2 Transmission in Alberta, British Columbia, and Ontario, Canada, December 25, 2019, to December 1, 2020

**DOI:** 10.1017/dmp.2021.78

**Published:** 2021-03-25

**Authors:** Isaac Chun-Hai Fung, Yuen Wai Hung, Sylvia K. Ofori, Kamalich Muniz-Rodriguez, Po-Ying Lai, Gerardo Chowell

**Affiliations:** 1 Department of Biostatistics, Epidemiology and Environmental Health Sciences, Jiann-Ping Hsu College of Public Health, Georgia Southern University, Statesboro, GA, USA; 2 Department of Health Sciences, Wilfrid Laurier University, Waterloo, ON, Canada; 3 Department of Biostatistics, School of Public Health, Boston University, Boston, MA, USA; 4 Department of Population Health Sciences, School of Public Health, Georgia State University, Atlanta, GA, USA

**Keywords:** Canada, coronavirus, COVID-19, epidemiology, infectious disease, SARS-CoV-2, transmission

## Abstract

**Objective:**

This study aimed to investigate coronavirus disease (COVID-19) epidemiology in Alberta, British Columbia, and Ontario, Canada.

**Methods:**

Using data through December 1, 2020, we estimated time-varying reproduction number, *R*_*t*_, using EpiEstim package in R, and calculated incidence rate ratios (IRR) across the 3 provinces.

**Results:**

In Ontario, 76% (92 745/121 745) of cases were in Toronto, Peel, York, Ottawa, and Durham; in Alberta, 82% (49 878/61 169) in Calgary and Edmonton; in British Columbia, 90% (31 142/34 699) in Fraser and Vancouver Coastal. Across 3 provinces, *R*_*t*_ dropped to ≤ 1 after April. In Ontario, *R*_*t*_ would remain < 1 in April if congregate-setting-associated cases were excluded. Over summer, *R*_*t*_ maintained < 1 in Ontario, ~1 in British Columbia, and ~1 in Alberta, except early July when *R*_*t*_ was > 1. In all 3 provinces, *R*_*t*_ was > 1, reflecting surges in case count from September through November. Compared with British Columbia (684.2 cases per 100 000), Alberta (IRR = 2.0; 1399.3 cases per 100 000) and Ontario (IRR = 1.2; 835.8 cases per 100 000) had a higher cumulative case count per 100 000 population.

**Conclusions:**

Alberta and Ontario had a higher incidence rate than British Columbia, but *R*_*t*_ trajectories were similar across all 3 provinces.

## Introduction

**Figure 1.**
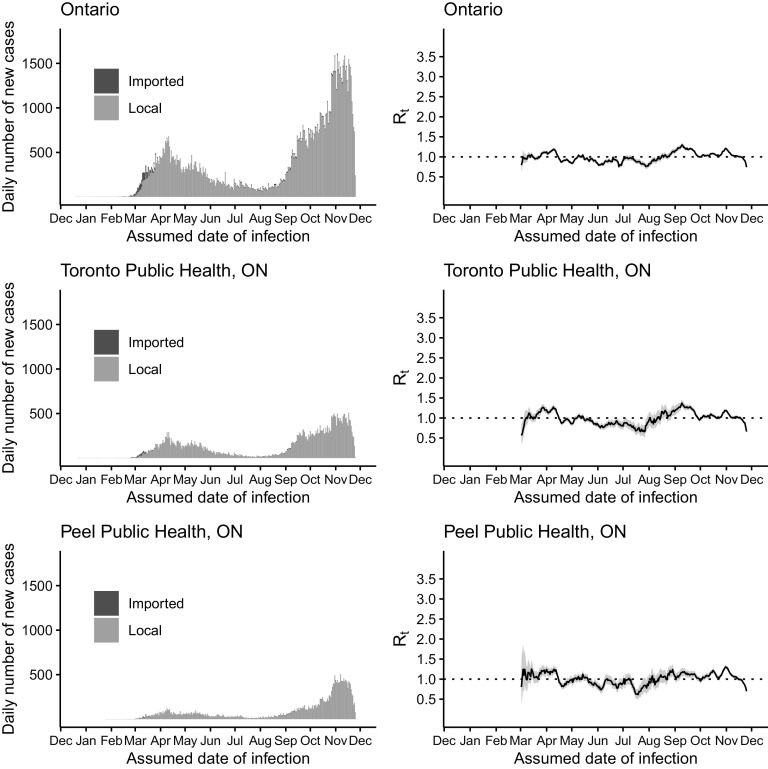
The daily number of new cases (left) and *R*_*t*_ (right) of COVID-19 in Ontario (upper panel), Toronto Public Health (middle panel), and Peel Public Health (lower panel) by the assumed date of infection from December 25, 2019, through December 1, 2020.

**Figure 2.**
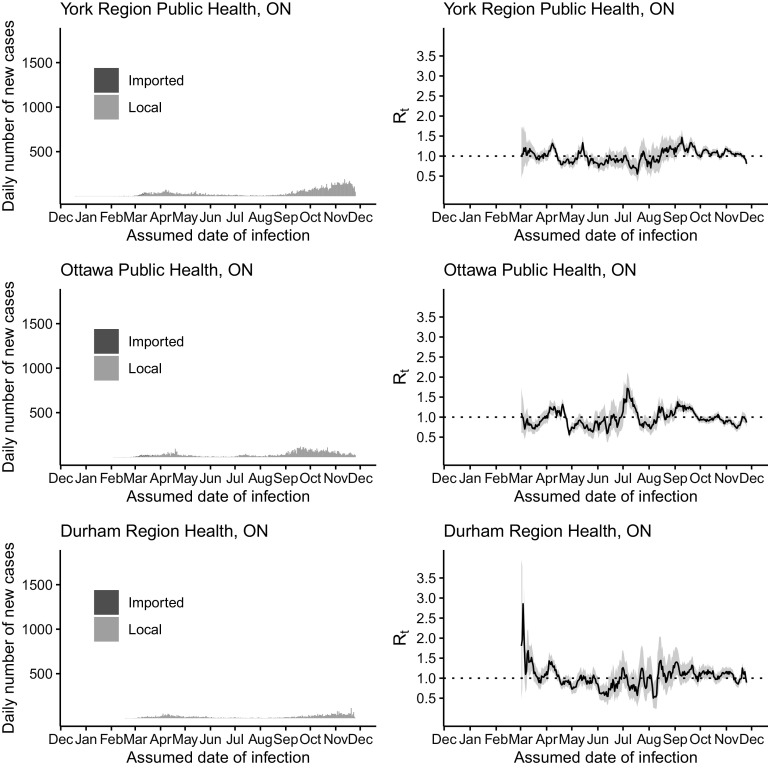
The daily number of new cases (left) and *R*_*t*_ (right) of COVID-19 in York Region Public Health (upper panel), Ottawa Public Health (middle panel), and Durham Public Health (lower panel) by the assumed date of infection from December 25, 2019, through December 1, 2020.

**Figure 3.**
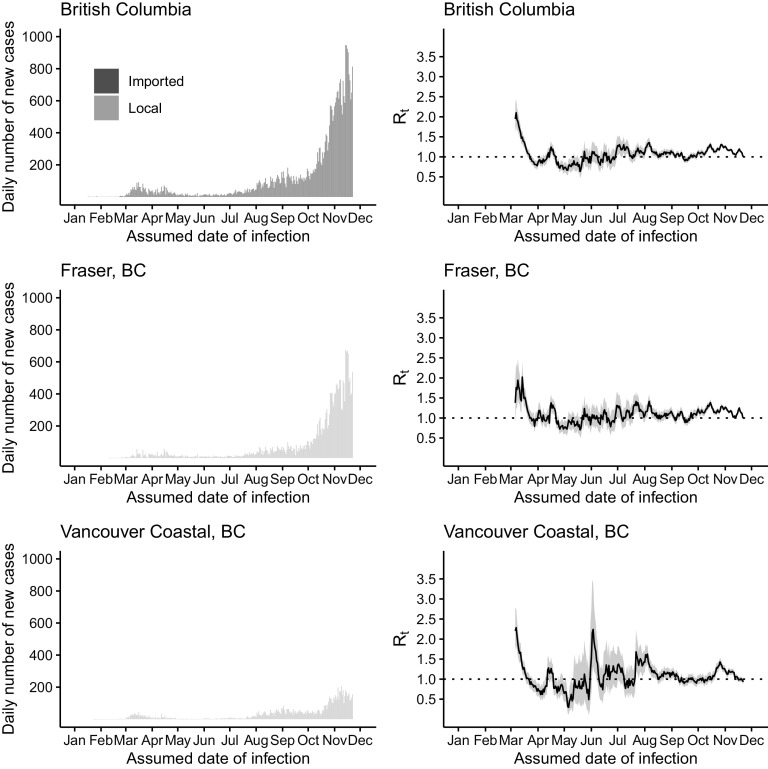
The daily number of new cases (left) and *R*_*t*_ (right) of COVID-19 in British Columbia (upper panel), Fraser (middle panel), and Vancouver Coastal (lower panel) by the assumed date of infection from January 1 through December 1, 2020.

**Figure 4.**
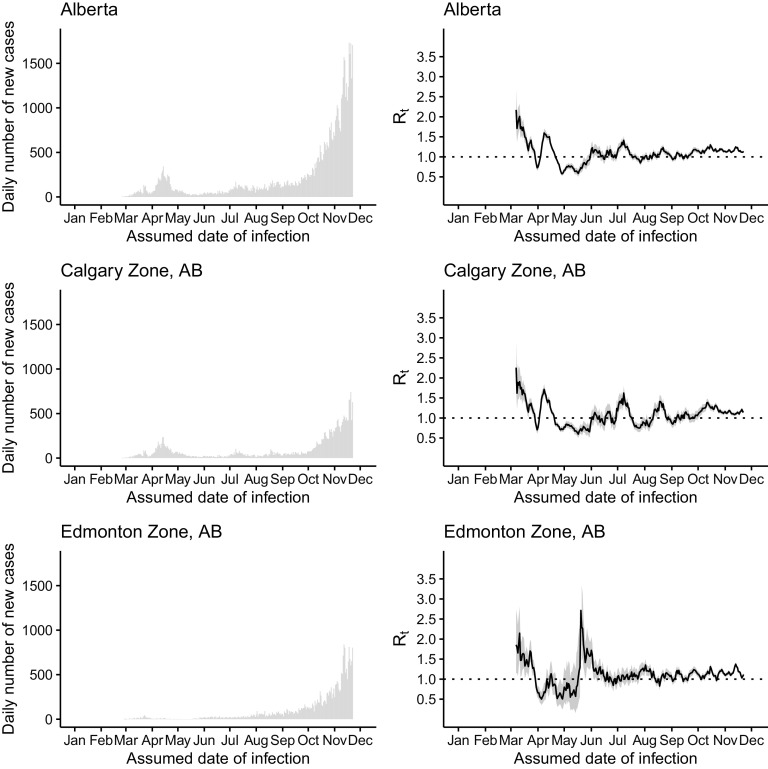
The daily number of new cases (left) and *R*_*t*_ (right) of COVID-19 in Alberta (upper panel), Calgary Zone (middle panel), and Edmonton Zone (lower panel) by the assumed date of infection from January 1 through December 1, 2020.

In 2020, the pandemic of the coronavirus disease 2019 (COVID-19), caused by severe acute respiratory syndrome coronavirus 2 (SARS-CoV-2), spread across Canada. The first imported case was presented in a Toronto hospital on January 23, 2020.^1,2^ On March 13, Quebec was the first province to declare a public health emergency^3^; 4 days later, Alberta, British Columbia, and Ontario also declared public health emergencies (Supplementary Table S1).^4^ As of December 14, a cumulative total of 468 862 cases, including 13 553 deaths, has been reported in Canada.^5^ While the Canadian epidemic trajectory appeared to have stabilized over summer, the case count has surged since October, as seen in Figures 1–4 for Alberta, British Columbia, and Ontario. In late 2020, province-wide restrictions have been re-introduced into Alberta (December 8, 2020),^6^ British Columbia (November 19, 2020),^7^ and Ontario (December 26, 2020).^8^

Epidemiologists have explored various aspects of the pandemic in Canada, including data availability,^9^ syndromic surveillance,^10^ disease burden and mortality,^11^ as well as the epidemiology in specific settings or subpopulations, such as nursing homes^12^ and intensive care units.^13^ Mathematical modelers have also made projections of epidemic trajectories to assist Ottawa and the provincial governments in their decision-making.^14-18^

This descriptive epidemiology study aimed to describe the COVID-19 epidemic in Alberta, British Columbia, and Ontario, Canada, from the first cases to December 1, 2020, by estimating and interpreting the time-varying reproduction number, *R*_*t*_, of SARS-CoV-2 in Alberta, British Columbia, and Ontario and select sub-provincial units, and comparing the cumulative case count per 100 000 across the 3 provinces.

## Methods

### Scope and Data Sources

We investigated the descriptive epidemiology and the *R*_*t*_ of SARS-CoV-2 in Alberta, British Columbia, and Ontario, and their public health subdivisions, using publicly available line lists of COVID-19 case data downloaded from provincial government websites. Alberta, British Columbia, and Ontario were chosen for this analysis given their line list data availability by sub-provincial unit (Supplementary Table S2). These data sets contained no personal identifiers to protect patients’ privacy. Population data by age and sex for Alberta, British Columbia, and Ontario were downloaded from the Alberta Government website.^19^ The Canadian Government’s national line list data set provided the accurate episode time in epidemiologic weeks instead of dates, and thus could not be analyzed using our method here. We selected the top few sub-provincial units that contributed to 75% or more of the cumulative case count of each province to be highlighted in this paper.

### Time Frame

The time frame of this study started on December 25, 2019 (the accurate episode date of the earliest confirmed case in York Region, Ontario), and ended on December 1, 2020, the last episode date (Ontario) or reported date (Alberta and British Columbia) in each of our data sets. Before we estimated the *R*_*t*_, we shifted the time series to approximate the date of infection. According to the US Centers for Disease Control and Prevention, the mean incubation period of COVID-19 is equal to 6 days and the median time lag from symptom onset to SARS-CoV-2 test is equal to 3 days.^20^ Therefore, Ontario data were shifted backward by 6 days as Ontario data were arranged by the accurate episode date (ie, date of symptom onset for those cases with symptom onset data). Likewise, Alberta and British Columbia data were shifted backward by 9 days as data were arranged by date of report.

The time frame for *R*_*t*_ estimation began with March 1, 2020. We did not estimate *R*_*t*_ in January and February 2020 because the small number of cases in Ontario and British Columbia during that time would lead to very uncertain *R*_*t*_ estimates. No cases were reported in Alberta before March 2020.

### Ontario Data Set

The Ontario data set was downloaded on December 3, 2020, at 2:18 PM (Eastern Standard Time, EST), and included cases until the end of December 1, 2020; 1 case reported on December 2, 2020, was dropped for consistency.^21^ In Ontario, the sub-provincial unit is a public health unit (PHU). There were 35 PHUs in Ontario,^22^ of which 34 PHUs had reported cases in this data set.

The Ontario data set contained the following variables: accurate episode date, case reported date, test reported date, specimen date, age group, gender, case acquisition information, clinical outcome, outbreak-related (*yes* or *no*), and the ID, name, address, city, postal code, website, latitude, and longitude of the reporting PHU.

In contrast to the Alberta and British Columbia data sets, which did not contain information on how the case-patients acquired the virus, the Ontario data set provided information on how a case-patient acquired the virus (“Case Acquisition Info”) (Supplementary Table S3). Among the 121 745 cases, 3205 (2.63%) were travel-related. In our *R*_*t*_ estimation, we categorized all travel-related cases as “imported” cases and all others as “local,” as per user instructions for the EpiEstim package. Please note that, to run the EpiEstim package, the first case(s) on the first day of a time series must be rendered as “imported” cases. Thus, if the first case(s) of a time series was not travel-related, we manually denoted them as “imported” cases so that *R*_*t*_ can be estimated using the EpiEstim package.

In the Ontario data set, there is another variable, “Outbreak-Related,” that indicates whether a case is associated with outbreaks in congregate settings, such as “long-term care home, retirement home, hospital, group home, shelter, correctional facility,” and others.^21^ In total, 30 438 (25%) of 121 745 cases were associated with COVID-19 outbreaks in congregate settings (Supplementary Tables S3 and S4). A sensitivity analysis was conducted in which cases associated with congregate settings are excluded (Supplementary Figures S1 and S2).

### Alberta Data Set

The Alberta data set was downloaded on December 3, at 2:20 PM (EST), and included cases until the end of December 1, 2020.^23^ In Alberta, each sub-provincial unit is a health services zone. There were 5 zones that reported cases in this data set. The Alberta data set contained the following variables: date of report, health services zone, gender, age group, case status (clinical outcome), and case type (confirmed or probable).

### British Columbia Data Sets

The British Columbia data set was downloaded on December 3, at 2:23 PM (EST), and included cases until the end of December 1, 2020; 29 cases reported on December 2, 2020, were excluded for consistency.^24^ In British Columbia, each sub-provincial unit is a health authority. There were 5 health authorities that reported cases in this data set. The British Columbia data set contained the following variables: date of report, health authorities, sex, age group, and classification reported (laboratory-diagnosed or “epidemiologically linked to another case,”^25^ ie, probable case). In the British Columbia data set, there is a health authority variable. According to the data notes,^25^ as of July 9, 2020, the variable represents the residence of the case, and if such information was unavailable, cases were assigned to the health authority reporting the case. For those case patients whose primary residence was overseas, they were marked as “Out of Canada.” In our *R*_*t*_ analysis, we categorized all “Out of Canada” cases as “imported” cases and all others as “local” cases, while we acknowledged that a case patient whose primary residence was in Canada might have been exposed to SARS-CoV-2 overseas.

We also downloaded the daily aggregate laboratory information on December 18, 2020, at 2:29 PM (EST), from British Columbia Centre for Disease Control,^24^ to generate the positivity rate plots for British Columbia, Fraser, and Vancouver Coastal (Supplementary Figure S3). Similar data were not available in machine-readable forms for Alberta and Ontario. However, trajectories of positivity rates that were available on Alberta’s official website^23^ and the official epidemiological report of Ontario,^26^ were used as comparison.

### Time-Varying Reproduction Number

In contrast with the basic reproduction number, *R*_*0*_, which represents the average number of secondary cases generated by an infectious individual in a totally susceptible population in the absence of interventions or behavioral changes, *R*_*t*_ is a time-varying indicator that represents the average number of secondary cases per infectious individual in a population as the epidemic unfolds in the presence of interventions and behavioral changes. *R*_*t*_ > 1 indicates sustained transmission and epidemic growth; *R*_*t*_ < 1 indicates unsustainable transmission and epidemic decline. As the epidemic runs its course, it is possible to quantify *R*_*t*_ over time after accounting for reductions in susceptibility in the population as more people acquire immunity through natural infection, behavior changes in the population, and interventions that mitigate the transmission rate.^27^

### Instantaneous Reproduction Number Method Implemented in R Package EpiEstim

There are multiple statistical methods available for *R*_*t*_ estimation,^28,29^ one of which is the instantaneous reproduction number method implemented in the R package EpiEstim.^30,31^ This method is considered to be well-suited to a near-real-time estimation of *R*_*t*_, and is sensitive to signals of changes in transmission potential given recent implementation and cessation of interventions or behavioral changes.^28^ This method has been applied in various studies to estimate the *R*_*t*_ of SARS-CoV-2 in different locations globally.^32-39^ A summary of the instantaneous reproduction number method is provided in the Online Supplementary Materials.

### Incidence Rate Ratio

The incidence rate (cumulative case count per 100 000 population) was calculated by dividing the cumulative number of cases in a province by its total population in 2019. Given that the time frame of our data sets is essentially the same, we did not use person-time as the denominator. We used British Columbia as the reference, given that the case count per 100 000 in British Columbia was the lowest among the 3 provinces, and we calculated the incidence rate ratio (IRR) of Alberta and that of Ontario by gender and age group. We did not calculate the 95% confidence interval for the estimates, because the estimates were true population estimates and not sample estimates, as we used data from province-wide surveillance systems that cover everyone in the province.

### Software Version

Data management and analysis (except for maps) was performed using Microsoft Excel and R version 3.6.2 (R Core Team, Vienna, Austria). Version 2.2-3 of the EpiEstim package was used. Maps were made in R version 3.5.1 (R Core Team, Vienna, Austria).

## Results

**Figure 5.**
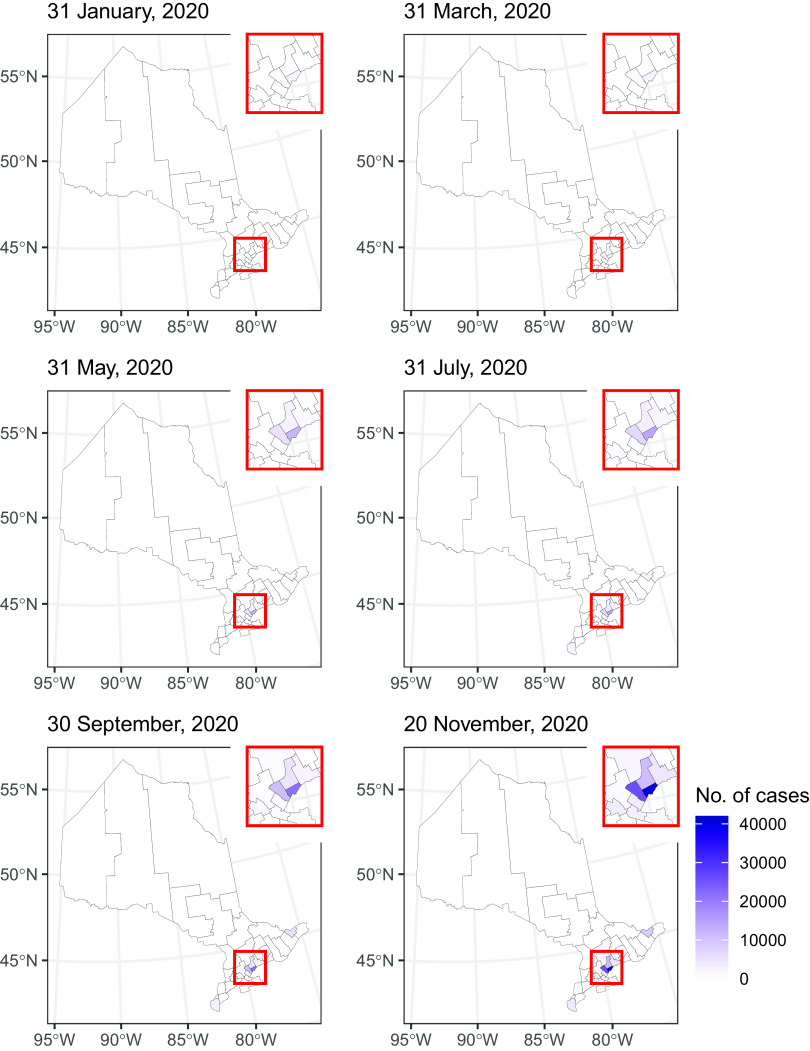
Map of the cumulative number of cases in Ontario by public health unit.

**Figure 6.**
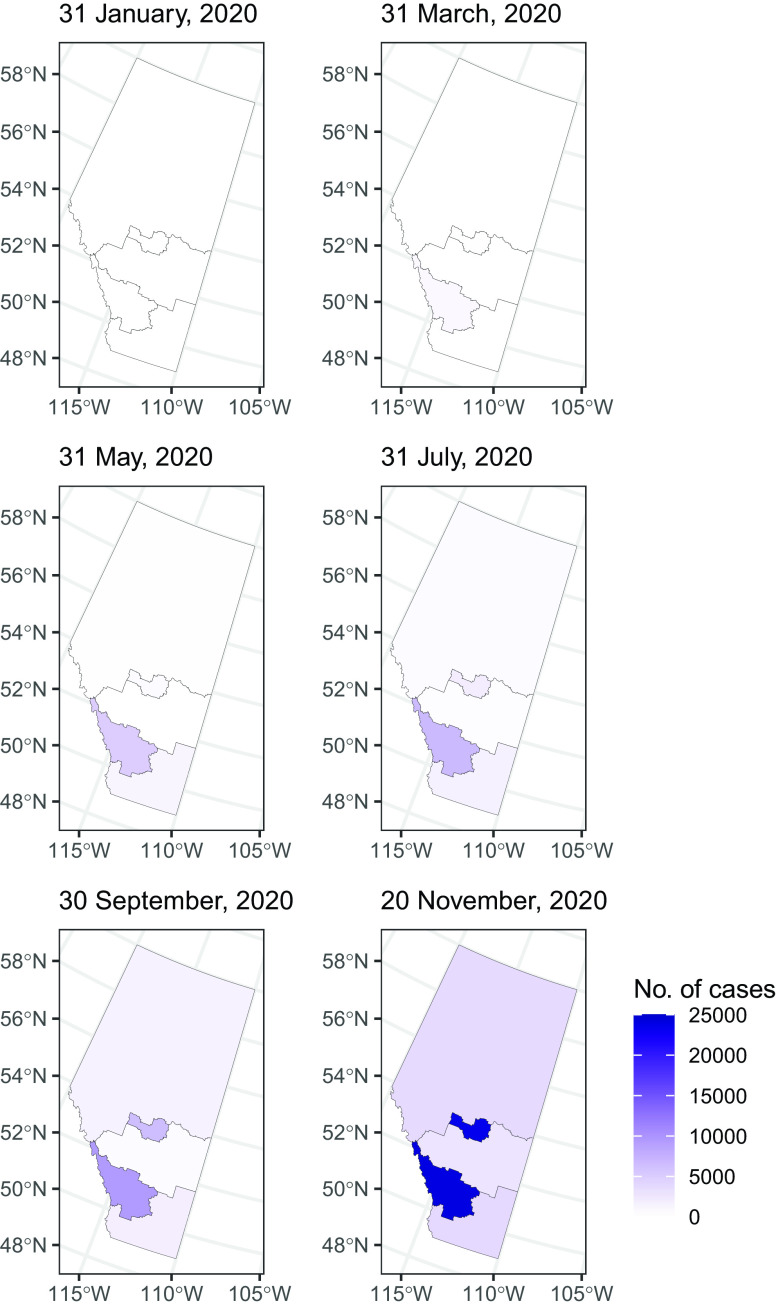
Map of the cumulative number of cases in Alberta by health service zone.

**Figure 7.**
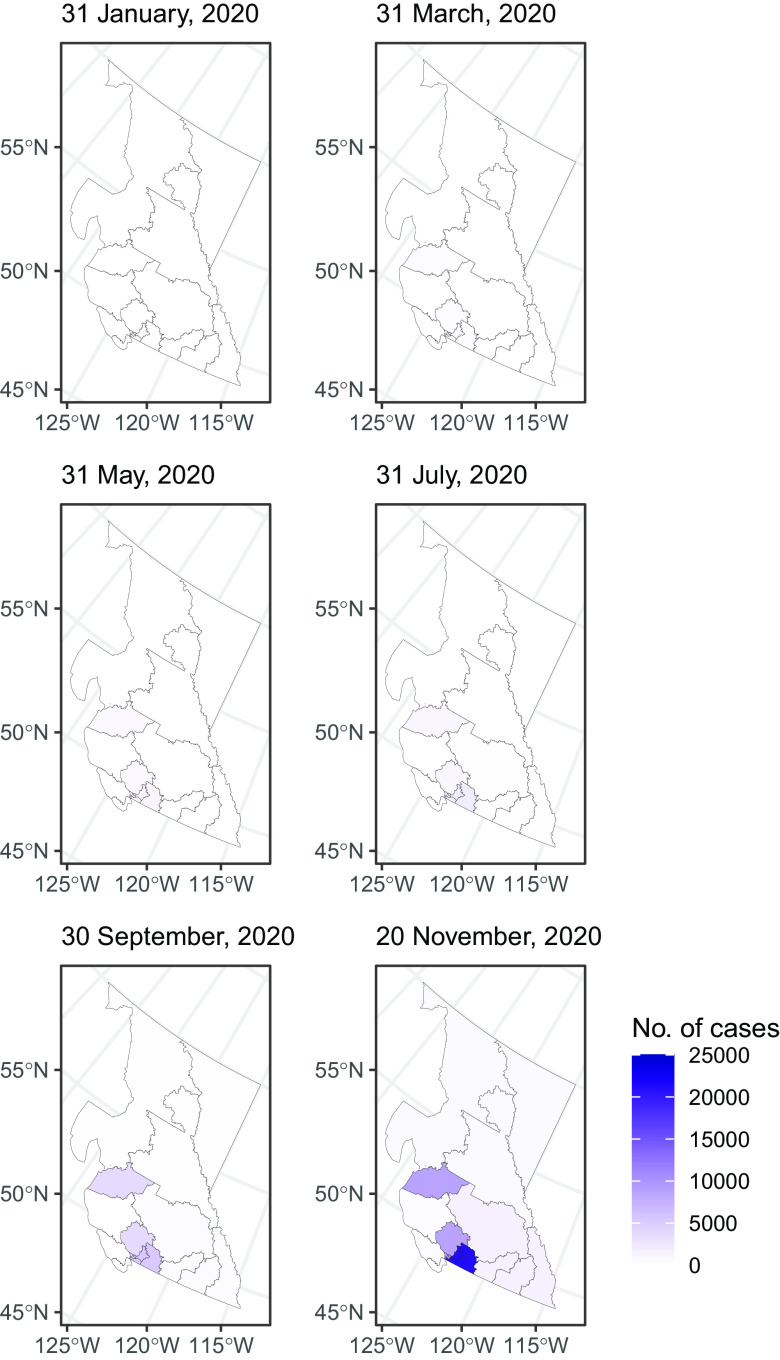
Map of the cumulative number of cases in British Columbia by health authority.

The dates of the first cases and the total number of cases in our data sets by each sub-provincial PHU in Alberta, British Columbia, and Ontario are presented in Supplementary Table S2. In early 2020, the epidemic was characterized by importation of cases from overseas. It was followed by community transmission while social distancing measures were gradually introduced. March 17 was the day when the public health emergency was declared in Alberta, British Columbia, and Ontario.^4^ Some mandatory social distancing measures were enforced from March through December. Provinces reopened in summer, but they were shut down again in November and December (Supplementary Table S1). Sub-provincial units with the highest case count that together contributed 75% or more cases to the province were highlighted in Figures 1, 2, 4, and 6. Five PHUs, namely, Toronto, Peel, York Region, Ottawa, and Durham Region (Supplementary Table S2), contributed 76% (92 745/121 745) of cases in Ontario (see Figures 1 and 2). Two zones, Calgary and Edmonton, contributed 82% (49 878/61 169) of cases in Alberta (Figure 3). Two health authorities, Fraser and Vancouver Coastal, contributed 90% (31 142/34 699) of cases in British Columbia (see Figure 4). Cumulative incidences over time were displayed on maps of Ontario (Figure 5), Alberta (Figure 6), and British Columbia (Figure 7).

### *Epidemic Curve and R*_*t*_

In Ontario, the majority of the imported cases were found in February and March 2020. On March 18, 2020, Canada closed the border to non-essential travels from overseas, including the United States (Supplementary Table S1). Henceforth, the number of imported cases quickly diminished (see Figure 1). The peak of the epidemic curve in April 2020 corresponded to an elevated *R*_*t*_ in late March and early April. *R*_*t*_ remained at a low level (around or below 1) over summer and elevated to above 1 from late August to November. Similar trends in *R*_*t*_ were observed in Toronto, Peel, York, Ottawa, and Durham, with some exceptions. For example, in York Region, *R*_*t*_ was elevated to > 1 in May. In Ottawa, there was an elevation of *R*_*t*_ > 1.5 in early July (see Figures 1-2).

As a sensitivity analysis, we repeated our analysis of Ontario and 5 PHUs in Toronto, Peel, York, Ottawa, and Durham, excluding cases associated with outbreaks in congregate settings. The *R*_*t*_ trajectories for these jurisdictions (Figures S1 and S2) were similar to those that included cases associated with congregate settings (Figures 1 and 2). The major exception was late March and early April, when *R*_*t*_ was > 1 if cases associated with congregate settings were included, and *R*_*t*_ was < 1 if they were excluded. This was consistent for both Ontario as a province and sub-provincial PHUs of Toronto, Peel, York, Ottawa, and Durham.

In Alberta, the peak of the incidence curve in April 2020 was primarily contributed by cases in Calgary, the major city in the province (see Figure 3). Both Alberta as a whole and Calgary, in particular, experienced an elevation of *R*_*t*_ to ~1.5 in April. In Alberta, sustained transmission over summer was reflected in *R*_*t*_ values that were around 1, with an elevation to around 1.3–1.4 in early July, apparently following Canada Day (July 1). This is also reflected in the *R*_*t*_ curve for Calgary. The sharp increase in *R*_*t*_ curve in Edmonton in the second half of May was due to the uncertainty around the *R*_*t*_ estimate as cases started to reappear in Edmonton after days of zero cases in the first half of May. The sustained increase in case count in Alberta from September to November was reflected in the *R*_*t*_ value that was elevated to > 1 during that period of time (see Figure 3).

In British Columbia, the outbreaks in March were reflected in an *R*_*t*_ estimate of ~2 (see Figure 4). However, this might be an overestimate, as some of the early cases might have been Canadians returning from overseas, and they were categorized as “local” in our analysis given their primary residences in British Columbia. Following a decrease in *R*_*t*_ to < 1 in late March, outbreaks in April led to an increase in *R*_*t*_ to > 1. *R*_*t*_ hovered around 1 over summer as sustained transmission continued at a low level in British Columbia. Since July, an *R*_*t*_ > 1 corresponds to sustained growth of the epidemic as daily incidence reached > 800 in November. The trajectory of *R*_*t*_ in Fraser is similar to that of the province. In Vancouver Coastal, the trajectory of *R*_*t*_ is similar to the province with an exception: The *R*_*t*_ peak in early June corresponded to the appearance of cases then after May when there were very few cases.

### IRR at the Provincial Level

Table S5 presents the number of cases per 100 000 population, stratified by sex/gender and age groups (see Tables S6–S8 for details). Interestingly, the incidence rate in Alberta was 2.0 times (1399.3/684.2) that of British Columbia, and that in Ontario was 1.2 times (835.8/684.2) that of British Columbia, which served as the reference group. Among females, the respective IRR in Alberta and Ontario were 2.1 (1390.6/662.4) and 1.3 (836.8/662.4). Among males, the respective IRR in Alberta and Ontario were 2.0 (1404.0/700.4) and 1.2 (824.6/700.2).

### Trajectory of Positive Rate in British Columbia

The trajectory of positivity rate of testing conducted in British Columbia (Figure S3) is similar to that of Alberta (as in Alberta’s official website’s Figure 20),^23^ and that of Ontario (as in the official Ontario epidemiological report’s Figure 3).^26^

## Discussion

Alberta, British Columbia, and Ontario, Canada, appeared to have managed to achieve limited transmission of SARS-CoV-2 with *R*_*t*_ of ~1 in summer 2020. However, all 3 provinces experienced a surge in case count in the autumn and winter of 2020 (see Figures 1–4). Our results show that, since mid-March in the 3 Canadian provinces under study, in the presence of social distancing and other public health interventions, SARS-CoV-2 has been spreading with an *R*_*t*_ < 2 that is smaller than the estimated *R*_*0*_ of > 2.^40,41^ During May to August, *R*_*t*_ fluctuated around 1. For Ontario, *R*_*t*_ has been consistently > 1 since late August. The drop in *R*_*t*_ to < 1 in late November was due to incomplete reporting of cases in the last 14 days of the daily time series as some infected individuals were in their incubation period. For Alberta and British Columbia, *R*_*t*_ has been > 1 since October. Our *R*_*t*_ estimates by sub-provincial unit present a similar picture as our province-level analysis, with few exceptions (see Figures 1–4). For example, in York Region, Ontario, *R*_*t*_ was elevated to > 1 in May due to a modest increase in case count. In Ottawa, Ontario, there was an elevation to *R*_*t*_ > 1.5 in early July as case count increased in July after a very low case count in June (see Figure 2). The sharp increase in *R*_*t*_ curve in Edmonton, Alberta, in the second half of May, was due to the uncertainty around the *R*_*t*_ estimate as cases started to reappear in Edmonton after days of zero cases in the first half of May (see Figure 3). In Vancouver Coastal, British Columbia, the peak of *R*_*t*_ in early June corresponded to reappearance of cases in early June, after May when there were very few cases (see Figure 4).

As in the United States,^42,43^ there had been outbreaks in long-term care facilities in Canada.^12,44,45^ We included a sensitivity analysis of the Ontario data set by excluding cases associated with congregate settings. The *R*_*t*_ trajectories with and without cases associated with congregate settings were similar, except for spring 2020 (Figures S1–S2). Apparently, cases associated with outbreaks in congregate settings have driven the *R*_*t*_ upward during the first wave of the COVID-19 pandemic in Ontario.^46^

While Canadian provinces have substantial administrative authority over the COVID-19 response, the federal and provincial governments have collaborated to produce a coordinated response to the pandemic. This is evidenced by the similar COVID-19 response measures in the 3 provinces, as well as the united politicians’ communications on the nature and severity of the COVID-19 pandemic across the different Canadian political parties.^47^ As the 3 provinces began reopening businesses, services, and public spaces, all 3 provinces have been gradually relaunching by stages which depended on local infection rate and the capacity of the health care system.^48-50^ Canada’s experience with the SARS epidemic in 2003 may have contributed to the coordinated government’s response with COVID-19. The 2003 SARS epidemic in Canada resulted in a set of recommendations to federal, provincial, and territorial leaders, and, in particular, led to the creation of the Public Health Agency of Canada, which has been leading the response to COVID-19.^51^ Given the consistency of these guidelines and policies across the 3 provinces (Table S1), it is surprising to observe that the cumulative case count per 100 000 population is higher in Alberta and Ontario than in British Columbia. We attempted to find an explanation through looking at the positivity rate of SARS-CoV-2 viral tests across these 3 provinces. However, the positivity rate trajectories were similar across these 3 provinces (Figure S3). Thus, the hypothesis that these are purely an artifact due to different testing strategies across the 3 provinces might not hold. However, we also did not identify a reason that can satisfactorily explain the difference in the cumulative case count per 100 000 population across the 3 provinces.

Our study has several limitations. First, our *R*_*t*_ estimation for Alberta and British Columbia relied on data by the reporting date and not the date of symptom onset, as the latter was unavailable to us. We shifted the data by 9 days to approximate the date of infection. Ontario provided the accurate episode date (primarily the date of symptom onset), and we shifted the data by 6 days to approximate the date of infection. Even though this is an approximation, a simple shift backward by the mean incubation period and the median delay to testing was found by Gostic et al. to be a simple and yet robust method to adjust the date of data to estimate *R*_*t*_.^28^ Second, the number of reported cases per day in the 3 data sets would be influenced by the testing rate, since limited testing capacity could lead to underdiagnosis. We did not attempt to do a nationwide analysis for Canada because the testing criteria have not been consistent across Canadian provinces. However, the trajectory of the positivity rate was similar across the 3 provinces studied herein. Third, our estimates are contingent upon the assumption that the testing ratio does not change significantly over the study period. However, provincial governments might have been ramping out testing capacity during the early epidemic phase in their jurisdiction. The trajectories of the positivity rate across the 3 provinces did vary over time, but they followed similar patterns. Fourth, reporting delays are known to vary over time and could influence the observed trajectory of the epidemics by date of report in Alberta and British Columbia. For Ontario, our results showed that the time lag from symptom onset to specimen collection, testing report, and case report has been reduced modestly over time (Online Supplementary Materials, Figures S4–S6). It is possible that similar improvement in reduction in testing delay may have happened in Alberta and British Columbia, but we do not have such data. Fifth, improvement in testing over time could potentially reduce generation time over time. As we do not have data on infector–infectee pairs, we cannot estimate whether the serial interval (which serves as proxy for generation time) has changed over time. In our *R*_*t*_ estimation, our serial interval distribution is parametrically determined *a priori* based on the literature.^52^ Sixth, for all 3 provinces, data for the last 2 weeks prior to the data download were lower than what they should have been, because there were cases that had not yet been reported to the provincial governments. Thus, the low *R*_*t*_ estimate for all the jurisdictions studied by the end of the time series is an artifact due to right-censoring of the data.

## Conclusions

In conclusion, up to December 1, 2020, 76% (92 745/121 745) of Ontario cases were in Toronto, Peel, York, Ottawa, and Durham; in Alberta, 82% (49 878/61 169) were in Calgary and Edmonton; in British Columbia, 90% (31 142/34 699) were in Fraser and Vancouver Coastal. *R*_*t*_ trajectories were similar across all 3 provinces. *R*_*t*_ dropped to ≤ 1 after April in all 3 provinces. In Ontario, *R*_*t*_ would remain < 1 in April if congregate-setting-associated cases were excluded. Over summer, *R*_*t*_ maintained < 1 in Ontario, ~1 in British Columbia, and ~1 in Alberta, except early July when *R*_*t*_ was > 1. In all 3 provinces, *R*_*t*_ was > 1, reflecting surges in case count from September through November. Compared with British Columbia (662.4 female and 700.3 male cases per 100 000), Alberta (female: IRR = 2.1, 1390.6/100 000; male: IRR = 2.0, 1404.0/100 000) and Ontario (female: IRR = 1.3, 836.8/100 000; male: IRR = 1.2, 824.6/100 000) had a higher cumulative case count per 100 000 population.

## Supplementary material

For supplementary material accompanying this paper visit http://dx.doi.org/10.1017/dmp.2021.78. click here to view supplementary material
