# Supplementary material for: SARS-CoV-2 Transmission in Alberta, British Columbia, and Ontario, Canada, December 25, 2019, to December 1, 2020
Source: Disaster Med Public Health Prep. 2021 Mar 25:1–10. doi: 10.1017/dmp.2021.78 (PMC8134904; doi:10.1017/dmp.2021.78)
Supplement: Supplementary file 1 [file dmpsup.zip › S1935789321000781sup001.docx]

**SARS-CoV-2 Transmission in Alberta, British Columbia, and Ontario, Canada, December 25, 2019 — December 1, 2020**

**Online Supplementary Materials**

**Authors:** Isaac Chun-Hai Fung, PhD; Yuen Wai Hung, PhD; Sylvia K. Ofori, MPH; Kamalich Muniz-Rodriguez, DrPH, MPH; Po-Ying Lai, MS; Gerardo Chowell, PhD.

**Affiliations:** Department of Biostatistics, Epidemiology and Environmental Health Sciences, Jiann-Ping Hsu College of Public Health, Georgia Southern University, Statesboro, Georgia, USA (Ms. Ofori, Dr. Muniz-Rodriguez, Dr. Fung); Department of Health Sciences, Wilfrid Laurier University, Waterloo, Ontario, Canada (Dr. Hung); Department of Biostatistics, School of Public Health, Boston University, Boston, Massachusetts, USA (Ms. Lai); Department of Population Health Sciences, School of Public Health, Georgia State University, Atlanta, Georgia, USA (Dr. Chowell)

Correspondence should be addressed to Dr. Isaac Chun-Hai Fung, Department of Biostatistics, Epidemiology and Environmental Health Sciences, Jiann-Ping Hsu College of Public Health, P.O. Box 7989, Georgia Southern University, Statesboro, GA 30460-7989, USA. Tel: +1 912-478-5079. Email: [cfung@georgiasouthern.edu](mailto:cfung@georgiasouthern.edu)

**Author Contributions.** Dr. Fung is the first and corresponding author. Dr. Hung, Ms. Ofori, Dr. Muniz-Rodriguez and Ms. Lai contributed equally as co-second authors. Dr. Chowell serves as the senior author.

**Additional details on the Methods**

***Further details about the Ontario dataset***

According to the data dictionary of the Ontario dataset, the variable for Case Acquisition Info (“Case_AcquisitionInfo”), i.e. the suspected method of exposure to COVID-19, if known, contains the following variables:^1^

| As of June 17, 2020 | As of October 6, 2020 |
| --- | --- |
| Close contact | Close contact |
| No Epi-link | No known epi link |
| No Info-Missing | Missing Information |
| No Info-Unknown | Unspecified epi link |
| Outbreak | Outbreak |
| Travel | Travel |

In the dataset that we analyzed, the category labels followed the updated terminology (as of October 6, 2020).

***Instantaneous reproduction number method implemented in R package EpiEstim***

We summarized the instantaneous reproduction number method below, of which detailed description has been made available elsewhere.^2,3^ The instantaneous reproduction number is the ratio between the number of incident cases at time *t*, and the total infectiousness of all the infected individuals at time t: *R_t_* = *I_t_*/*Λ_t_*, where $\Lambda_{t}=\sum_{s=1}^{t} I_{t-s}w_{s}$. The total infectiousness is the sum of infection incidence up to the previous time point (*t-1*) weighted by an infectivity function *w_s_*. The infectivity function is a probability distribution that describes the average infectiousness profile of an individual after being infected, and it is usually represented by the serial interval distribution as an approximation to the generation time distribution. The number of new cases at time *t* is therefore a Poisson distribution with a mean of $R_{t}\sum_{s=1}^{t} I_{t-s}w_{s}$. Given the reproduction number *R_t_*, and conditional on the time series of daily new cases, *I_0_*, …, *I_t-1_*, the likelihood of the incidence *I_t_* is therefore, $\left( I_{t}|I_{0},\ldots,I_{t-1},w,R_{t} \right)=\frac{\left( R_{t}\Lambda_{t} \right)^{I_{t}}e^{-R_{t}\Lambda_{t}}}{I_{t}!}$ where $\Lambda_{t}=\sum_{s=1}^{t} I_{t-s}w_{s}$. Therefore, $R_{t}=I_{t}/(\sum_{s=1}^{t} I_{t-s}w_{s}).$ We can interpret the instantaneous reproduction number as the average number of secondary cases that an infectious person will infect at time *t*, if conditions in previous time points remain the same at time *t*. Given this formulation of the instantaneous reproduction number, *R_t_* is highly sensitive to fluctuation in the incidence data. To minimize *R_t_* variation, the EpiEstim package allows user to specify a time window over which the *R_t_* estimate is assumed to be constant. The window is of size *τ* and ends at time *t*. If transmissibility is assumed constant over time from (*t*-*τ*+1) to *t*, and is denoted by a reproduction number, *R_t,τ_*, the likelihood of the incidence during the time period, from *I_(t-τ+1)_* to *I_t_*, conditional on incidence prior to the time period, from *I_0_*, to *I_(t-τ)_*, is found to be:^2^

$$P\left( I_{t-\tau+1},\ldots,I_{t} | I_{0},\ldots,I_{t-\tau},w,R_{t,\tau} \right)=\prod_{s=t-\tau+1}^{t} [\frac{\left( R_{t,\tau}\Lambda_{s} \right)^{I_{s}}e^{-R_{t,\tau}\Lambda_{s}}}{I_{s}!}]$$

The EpiEstim package utilized a Bayesian framework with a Gamma-distributed prior for *R_t,τ_*, and the analytical expression of the posterior distribution of *R_t_* and estimated its median, the variance and the 95% credible interval (CrI).^2^

Given the small number of cases in January and February and thus wide uncertainty bounds of the *R_t_* estimates, we presented *R_t_* estimates from March to November (by the date of assumed infection). *R_t_* estimates of a 7-day time window, over which the *R_t_* estimates is assumed to be constant, were presented with their 95% CrI. We did not use a time window shorter than 7 days because such *R_t_* estimate would be very sensitive to daily incidence fluctuation. Furthermore, for surveillance data by date of report (Alberta and British Columbia), weekend effect is common given the 5-day work week, i.e., daily number of cases reported over Saturday and Sunday would be lower than those from Monday to Friday. Therefore, it is reasonable to use time-windows of 7 days.

Serial interval is assumed to follow a gamma distribution (mean: 4.60 days; standard deviation: 5.55).^4^

***Map creation.*** The health region boundaries shapefile was downloaded at Statistics Canada at https://www150.statcan.gc.ca/n1/pub/82-402-x/2018001/hrbf-flrs-eng.htm. The health regions were based on 2016 Census geographic units. Maps were made in R version 3.5.1 (R Core Team, Vienna, Austria). The boundaries shapefiles are transformed by R packages rnaturalearth (version 0.1.0) and sf (version 0.9.0), merged with the study data by health regions, and presented by R package ggplot2 (version 3.3.0). The maps show the cumulative number of cases by assumed date of infection on January 31, March 31, May 31, July 31, September 30 and November 20, 2020.

**Additional Results**

An analysis of the Ontario dataset found that the time lag between the specimen date and the accurate episode date of cases had a median of 1 day (interquartile range, 0, 4) and decreased over time by 0.02497 days per day (Figure S4). Likewise, the time lag between the test report date and the accurate episode date of Ontario cases had a median of 3 days (interquartile range, 2, 6) and decreased over time by 0.01568 days per day (Figure S5). Similarly, the time lag between the case report date and the accurate episode date of Ontario cases had a median of 3 days (interquartile range, 2, 6) and decreased over time by 0.009309 days per day (Figure S6).

**Figure S1.** Sensitivity analysis: Daily number of new cases and *R_t_* of cases in non-congregate settings in Ontario, and Toronto and Peel health regions, by assumed date of infection.


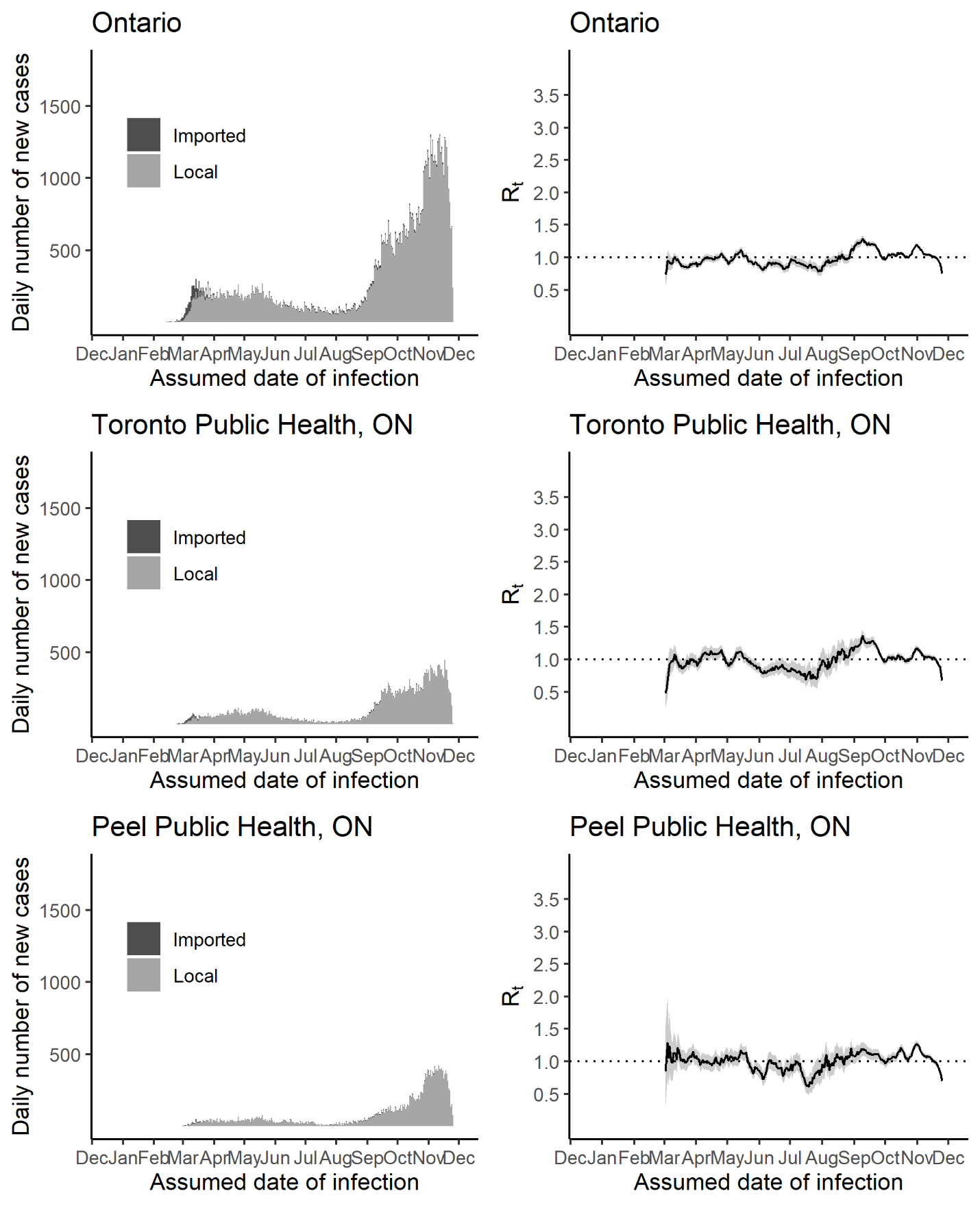


**Figure S2.** Sensitivity analysis: Daily number of new cases and *R_t_* of cases in non-congregate settings, in York, Ottawa and Durham health regions, Ontario, by assumed date of infection.


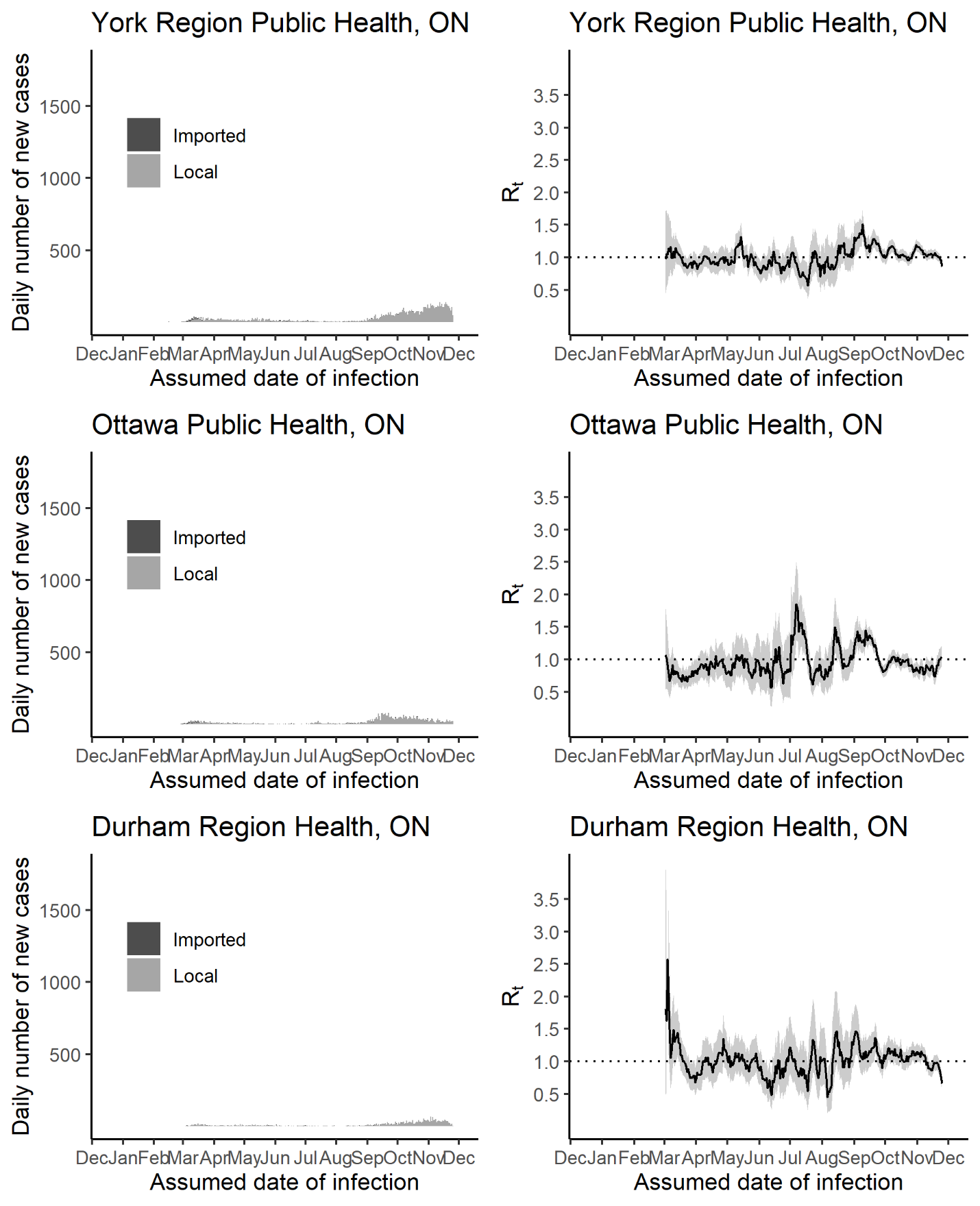


**Figure S3.** Positivity rate of SARS-CoV-2 viral test in British Columbia, as well as Fraser and Vancouver Coastal health authorities by date of report from January 23, 2020 to December 1, 2020.


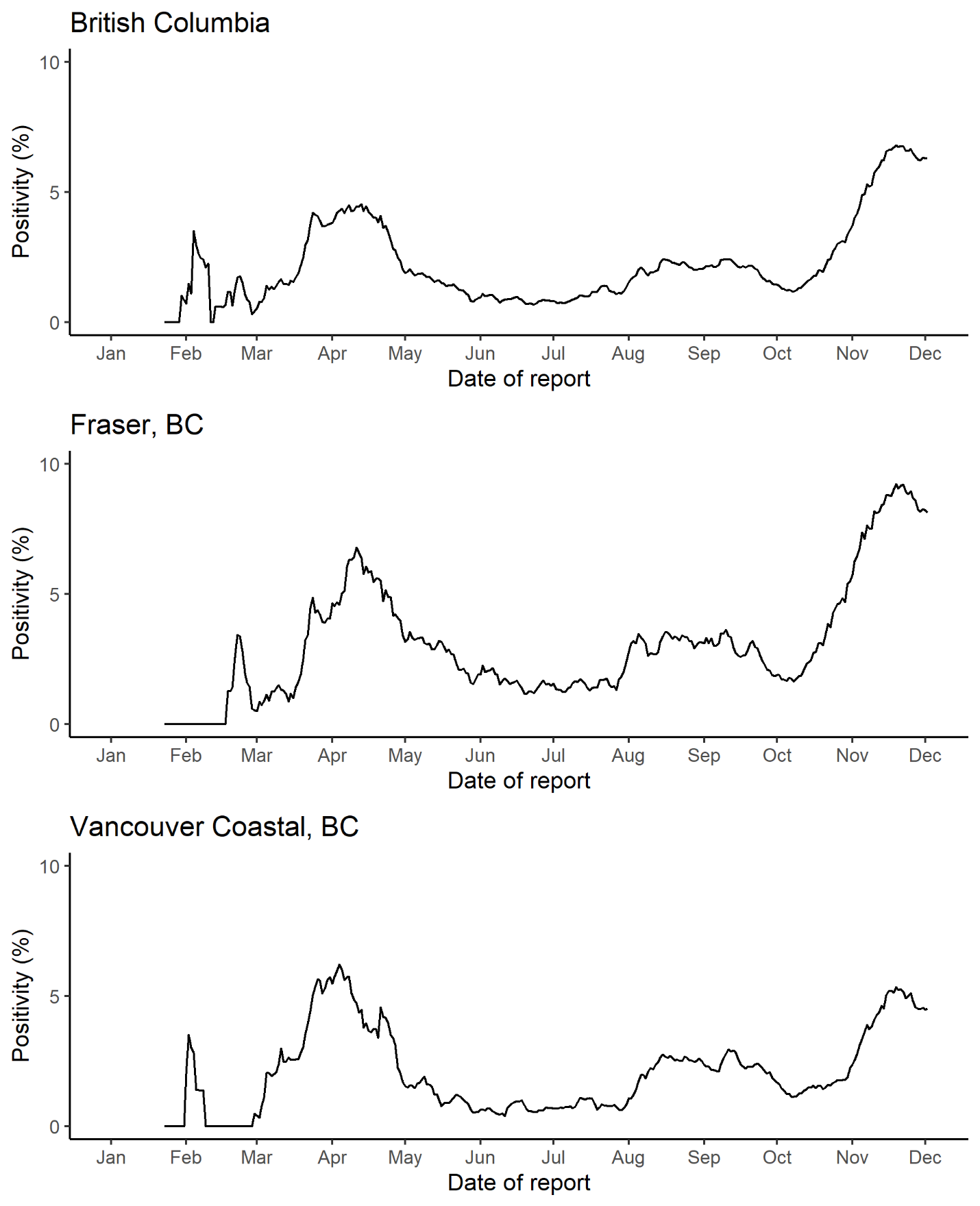


**Figure S4.** Scatter plot of the time difference between testing (specimen date) and symptom onset (accurate episode date) of each case-patient in Ontario over time (accurate episode date), December 25, 2019 – December 1, 2020 (showing 95,748 values, excluding 25,997 missing values). The red dashed regression line represents a regression line: delay-in-specimen-collection = 467.4 – 0.02497 accurate-episode-date. This translates into a 3.1-day delay on December 1, 2020.


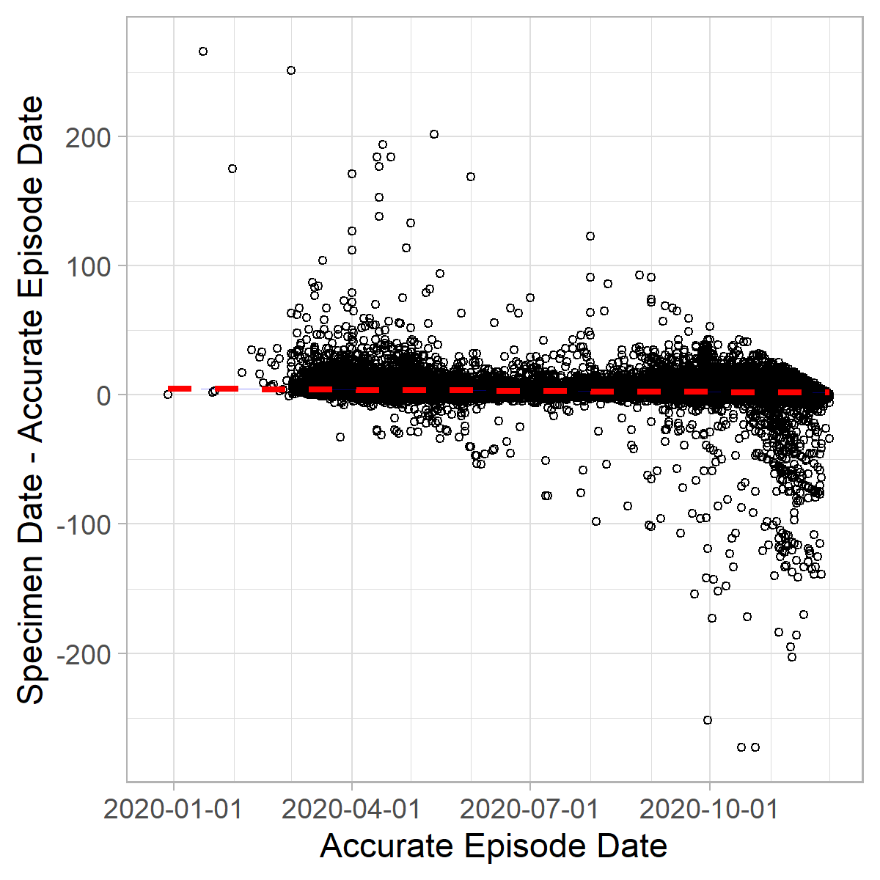


**Figure S5.** Scatter plot of the time difference between test report date and symptom onset (accurate episode date) of each case-patient in Ontario over time (accurate episode date), December 25, 2019 – December 1, 2020 (showing 120,061 values, excluding 1684 missing values). The red dashed regression line represents a regression line: delay-in-test-report = 174.7 – 0.009309 accurate-episode-date. This translates into a 1.6-day delay on December 1, 2020.


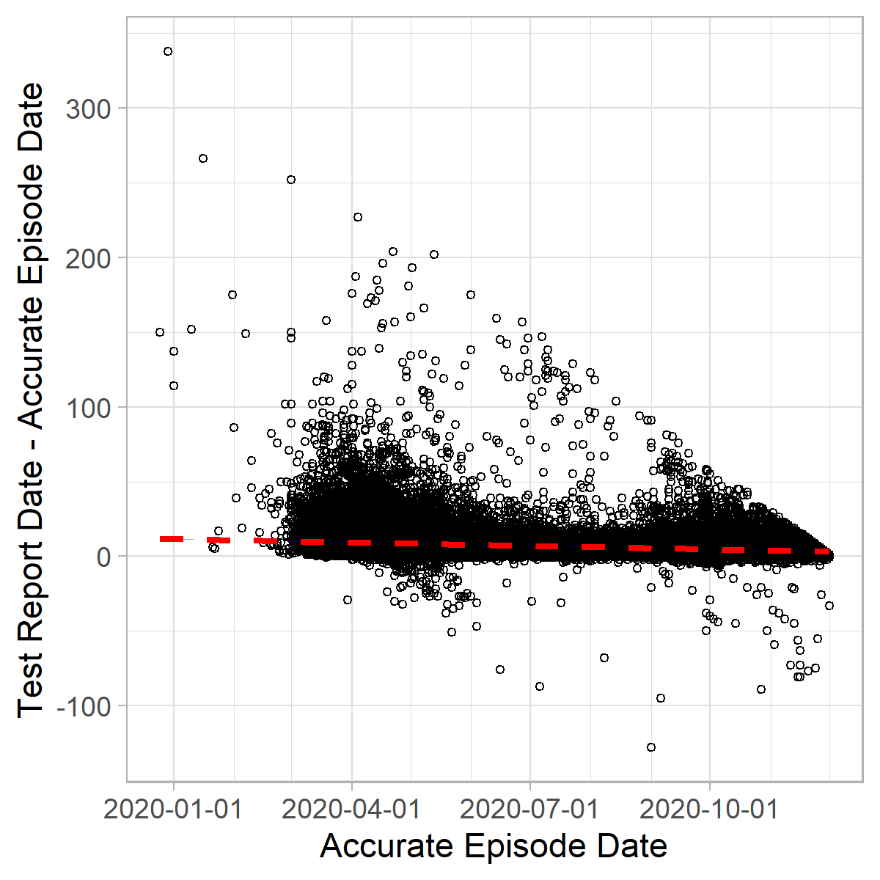


**Figure S6.** Scatter plot of the time difference between case report date and symptom onset (accurate episode date) of each case-patient in Ontario over time (accurate episode date), December 25, 2019 – December 1, 2020 (showing 121744 values, excluding 1 missing value). The red dashed regression line represents a regression line: delay-in-case-report = 295.1 – 0.01568 accurate-episode-date. This translates into a 3.6-day delay on December 1, 2020.


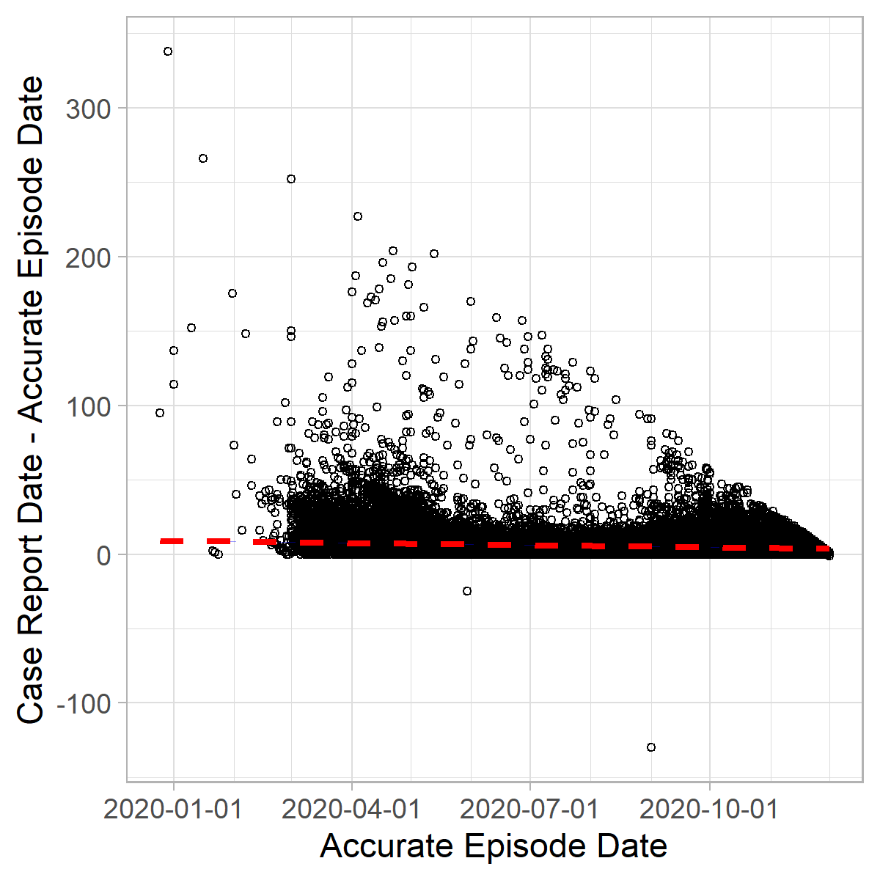


**References**

1. Government of Ontario. Confirmed positive cases of COVID-19 in Ontario. 2020; <https://data.ontario.ca/dataset/confirmed-positive-cases-of-covid-19-in-ontario>. Accessed December 3, 2020.

2. Cori A, Ferguson NM, Fraser C, Cauchemez S. A new framework and software to estimate time-varying reproduction numbers during epidemics. *Am J Epidemiol.* 2013;178(9):1505-1512.

3. Thompson RN, Stockwin JE, van Gaalen RD, et al. Improved inference of time-varying reproduction numbers during infectious disease outbreaks. *Epidemics.* 2019;29:100356.

4. You C, Deng Y, Hu W, et al. Estimation of the time-varying reproduction number of COVID-19 outbreak in China. *Int J Hyg Environ Health.* 2020;228:113555.

5. CTV News. Tracking every case of COVID-19 in Canada. 2020; <https://www.ctvnews.ca/health/coronavirus/tracking-every-case-of-covid-19-in-canada-1.4852102>. Accessed May 2, 2020.

6. Global News. Timeline: How Canada has changed since coronavirus was declared a pandemic. April 11 2020. . 2020; <https://globalnews.ca/news/6800118/pandemic-one-month-timeline/>. Accessed May 2, 2020.

7. Public Health Ontario. Coronavirus Disease 2019 (COVID-19) Testing. 2020; <https://www.publichealthontario.ca/en/laboratory-services/test-information-index/wuhan-novel-coronavirus>. Accessed May 2, 2020.

8. Government of Alberta. CMOH order 01-2020 : 2020 COVID-19 response. 2020; <https://open.alberta.ca/publications/cmoh-order-01-2020-2020-covid-19-response>. Accessed January 12, 2021.

9. The Global and Mail. Coronavirus March 17: Ontario, Alberta, B.C. declare states of emergency; B.C. closes schools to combat virus spread. 2020; <https://www.theglobeandmail.com/canada/article-the-latest-on-the-coronavirus-cineplex-closes-its-doors-a-quieter-st/>. Accessed April 7, 2020.

10. Government of Alberta. CMOH order 02-2020 : 2020 COVID-19 response. 2020; <https://open.alberta.ca/publications/cmoh-order-02-2020-2020-covid-19-response>. Accessed January 12, 2021.

11. Government of Alberta. CMOH order 03-2020 : 2020 COVID-19 response. 2020; <https://open.alberta.ca/publications/cmoh-order-03-2020-2020-covid-19-response>. Accessed January 12, 2021.

12. Government of Alberta. CMOH order 05-2020 : 2020 COVID-19 response. 2020; <https://open.alberta.ca/publications/cmoh-order-05-2020-2020-covid-19-response>. Accessed January 12, 2021.

13. Government of Alberta. CMOH order 28-2020 which amends CMOH order 05-2020 : 2020 COVID-19 response. 2020; <https://open.alberta.ca/dataset/cmoh-order-28-2020-which-amends-cmoh-order-05-2020-2020-covid-19-response>. Accessed January 12, 2021.

14. Government of Alberta. Clarification of CMOH order 05-2020 : COVID-19 response. 2020; <https://open.alberta.ca/publications/clarification-of-cmoh-order-05-2020>. Accessed January 12, 2021.

15. Global News. Alberta closes some non-essential businesses, prevents evictions as 542 COVID-19 cases confirmed. March 27, 2020. 2020; <https://globalnews.ca/news/6742251/alberta-health-coronavirus-covid-19-march-27/>. Accessed May 2, 2020.

16. Government of Alberta. CMOH order 07-2020 : 2020 COVID-19 response. 2020; <https://open.alberta.ca/publications/cmoh-order-07-2020-2020-covid-19-response>. Accessed January 12, 2021.

17. Vogel L. COVID-19: A timeline of Canada’s first-wave response. *CMAJ News* 2020; <https://cmajnews.com/2020/06/12/coronavirus-1095847/>. Accessed July 13, 2020.

18. Government of Alberta. CMOH order 09-2020 : 2020 COVID-19 response. 2020; <https://open.alberta.ca/publications/cmoh-order-09-2020-2020-covid-19-response>. Accessed January 12, 2021.

19. Government of Alberta. CMOH order 14-2020 which rescinds CMOH order 09-2020 : 2020 COVID-19 response. 2020; <https://open.alberta.ca/publications/cmoh-order-14-2020-2020-covid-19-response>. Accessed January 12, 2021.

20. Government of Alberta. CMOH order 15-2020 : 2020 COVID-19 response. 2020; <https://open.alberta.ca/publications/cmoh-order-15-2020-2020-covid-19-response>. Accessed January 12, 2021.

21. Government of Alberta. CMOH order 16-2020 which amends CMOH Order 07-2020 : 2020 COVID-19 response. 2020; <https://open.alberta.ca/publications/cmoh-order-16-2020-2020-covid-19-response>. Accessed January 12, 2021.

22. Government of Alberta. CMOH order 17-2020 : 2020 COVID-19 response. 2020; <https://open.alberta.ca/publications/cmoh-order-17-2020-2020-covid-19-response>. Accessed January 12, 2021.

23. Government of Alberta. CMOH order 18-2020, which rescinds CMOH order 04-2020 and modifies CMOH orders 01-2020, 02-2020 and 07-2020 : COVID-19 response. 2020; <https://open.alberta.ca/publications/cmoh-order-18-2020-rescinds-04-2020-and-modifies-orders-01-02-and-07-2020-covid-19-response>. Accessed January 12, 2021.

24. Government of Alberta. CMOH order 19-2020, which modifies CMOH orders 01-2020, 02-2020 and 07-2020 : COVID-19 response. 2020; <https://open.alberta.ca/publications/cmoh-order-19-2020-which-modifies-cmoh-orders-01-02-and-07-2020-covid-19-response>, January 12, 2021.

25. Government of Alberta. CMOH order 20-2020 which amends CMOH Order 07-2020 : 2020 COVID-19 response. 2020; <https://open.alberta.ca/publications/cmoh-order-20-2020-which-amends-cmoh-order-07-2020-2020-covid-19-response>. Accessed January 12, 2021.

26. Government of British Columbia. BC's Restart Plan. 2020; <https://www2.gov.bc.ca/gov/content/safety/emergency-preparedness-response-recovery/covid-19-provincial-support/bc-restart-plan>. Accessed July 13, 2020.

27. Government of Ontario. Archived - Reopening Ontario in stages. 2020; <https://www.ontario.ca/page/reopening-ontario-stages>. Accessed December 29, 2020.

28. Government of Alberta. CMOH order 24-2020, which modifies CMOH orders 18-2020 and 19-2020 : COVID-19 response. 2020; <https://open.alberta.ca/publications/cmoh-order-24>. Accessed January 12, 2021.

29. Government of Alberta. CMOH order 25-2020, which rescinds CMOH orders 01-2020, 02-2020, 07-2020, 15-2020, 16-2020, 17-2020, 18-2020, 19-2020, 20-2020 and 24-2020 : COVID-19 response. 2020; <https://open.alberta.ca/dataset/cmoh-order-25-2020-which-rescinds-cmoh-orders-01-02-07-15-16-17-18-19-20-24-2020-covid-19-response>. Accessed January 12, 2021.

30. CTV News Edmonton Staff. Alberta to lift state of public health emergency, 20 new COVID-19 cases confirmed. 2020; <https://edmonton.ctvnews.ca/alberta-to-lift-state-of-public-health-emergency-20-new-covid-19-cases-confirmed-1.4984901>. Accessed January 12, 2021.

31. Government of British Columbia. Phase 3 - BC's Restart Plan. 2020; <https://www2.gov.bc.ca/gov/content/safety/emergency-preparedness-response-recovery/covid-19-provincial-support/phase-3>. Accessed July 13, 2020.

32. Government of Alberta. CMOH order 26-2020 : COVID-19 response. 2020; <https://open.alberta.ca/publications/cmoh-order-26-2020-covid-19-response>. Accessed January 12, 2021.

33. Government of Alberta. CMOH order 29-2020 which rescinds CMOH order 14-2020 : 2020 COVID-19 response. 2020; <https://open.alberta.ca/publications/cmoh-order-29-2020-which-rescinds-cmoh-order-14-2020-2020-covid-19-response>. Accessed January 12, 2021.

34. Government of Alberta. CMOH order 33-2020 : 2020 COVID-19 response. 2020; <https://open.alberta.ca/publications/cmoh-order-33-2020-2020-covid-19-response>. Accessed January 12, 2021.

35. Government of Ontario. Reopening Ontario. 2020; <https://www.ontario.ca/page/reopening-ontario>. Accessed December 29, 2020.

36. Government of Alberta. CMOH order 34-2020 which amends CMOH Order 25-2020 : 2020 COVID-19 response. 2020; <https://open.alberta.ca/publications/cmoh-order-34-2020-which-amends-cmoh-order-25-2020-2020-covid-19-response>. Accessed January 12, 2021.

37. Government of Alberta. CMOH order 35-2020 : 2020 COVID-19 response. 2020; <https://open.alberta.ca/publications/cmoh-order-35-2020-2020-covid-19-response>. Accessed January 12, 2021.

38. Government of Alberta. CMOH order 36-2020 which rescinds CMOH order 35- 2020 : 2020 COVID-19 response. 2020; <https://open.alberta.ca/publications/cmoh-order-36-2020-which-rescinds-cmoh-order-35-2020-2020-covid-19-response>. Accessed January 12, 2021.

39. Government of Alberta. CMOH order 37-2020 : 2020 COVID-19 response. 2020; <https://open.alberta.ca/publications/cmoh-order-37-2020-2020-covid-19-response>. Accessed January 12, 2021.

40. Government of British Columbia. Province-wide restrictions. 2020; <https://www2.gov.bc.ca/gov/content/safety/emergency-preparedness-response-recovery/covid-19-provincial-support/restrictions>. Accessed December 29, 2020.

41. Government of Alberta. CMOH order 38-2020 which rescinds CMOH order 36- 2020 and part 3 of CMOH order 37-2020 : 2020 COVID-19 response. 2020; <https://open.alberta.ca/publications/cmoh-order-38-2020>. Accessed January 12, 2021.

42. Government of Alberta. CMOH order 39-2020 which rescinds part 1, 2 and 4 of CMOH order 37-2020 : 2020 COVID-19 response. 2020; <https://open.alberta.ca/publications/cmoh-order-39-2020>. Accessed January 12, 2021.

43. Government of Alberta. CMOH order 40-2020 which amends CMOH order 38-2020 and CMOH order 39-2020 : 2020 COVID-19 response. 2020; <https://open.alberta.ca/publications/cmoh-order-40-2020>. Accessed January 12, 2021.

44. Government of Alberta. Stronger public health measures. 2020; <https://www.alberta.ca/enhanced-public-health-measures.aspx>. Accessed December 29, 2020.

45. Government of Alberta. CMOH order 41-2020 which amends CMOH order 38-2020 : 2020 COVID-19 Response. 2020; <https://open.alberta.ca/publications/cmoh-order-41-2020>. Accessed January 12, 2021.

46. Government of Alberta. CMOH order 42-2020 which rescinds CMOH order 38-2020 and 39-2020 : 2020 COVID-19 Response. 2020; <https://open.alberta.ca/publications/cmoh-order-42-2020>. Accessed January 12, 2021.

47. Government of Alberta. CMOH order 44-2020 which amends CMOH order 42-2020 and rescinds CMOH Order 43-2020. 2020; <https://open.alberta.ca/publications/cmoh-order-44-2020>. Accessed January 12, 2021.

48. Government of Ontario. COVID-19: provincewide shutdown. 2020; <https://www.ontario.ca/page/covid-19-provincewide-shutdown>. Accessed December 29, 2020.

**Supplementary Tables**

**Table S1.** Timeline of first COVID-19 cases and public health measures taken in response to COVID-19 in Alberta, British Columbia, and Ontario in 2020.

| Date  (yyyy-mm-dd) | Events | Reference |
| --- | --- | --- |
| 2020-01-25 | First case of COVID-19 in Canada was reported in Toronto, Ontario. | ^5^ |
| 2020-01-28 | First presumptive case of COVID-19 was reported in British Columbia. | ^5^ |
| 2020-03-05 | First case of community transmission in British Columbia was detected through the annual influenza surveillance program; first presumptive case of travel-related COVID-19 was reported in Alberta. | ^5^ |
| 2020-03-12 | Ontario ordered public school closure. | ^6^ |
| 2020-03-15 | First community transmission case was reported in Alberta. | ^5^ |
| 2020-03-16 | Change in Public Health Ontario Laboratory testing algorithm of COVID-19. | ^7^ |
| 2020-03-16 | Alberta prohibited attendance at early childhood service programs, day cares, out of school care, preschool programs, schools, post-secondary institutions and other educational settings in Alberta (CMOH order 01-2020). | ^8^ |
| 2020-03-17 | Alberta, British Columbia and Ontario declared states of emergency due to COVID-19, ordered many businesses, including bars, cinemas and private schools, to close. Gathering of more than 50 people were prohibited in Alberta (CMOH order 02-2020). First COVID-19 related death was reported in Ontario | ^9,10^ |
| 2020-03-18 | Canada closed border to non-essential travels (including the United States). | ^6^ |
| 2020-03-19 | Alberta had its first death from COVID-19. | ^5^ |
| 2020-03-20 | Alberta imposed limitations to visitors at health care facilities (CMOH order 03-2020). | ^11^ |
| 2020-03-23 | Ontario ordered mandatory closure of non-essential businesses | ^6^ |
| 2020-03-25 | Alberta required any person who is a confirmed case of COVID-19 be in isolation for a minimum of 10 days from the start of their symptoms, or until symptoms resolve, and requires any person entering Alberta after having travelled internationally, or is a close contact of a confirmed case to quarantine for a minimum 14-day period (CMOH order 05-2020, modified by Order 28-2020 on July 3, 2020 and clarified on August 27, 2020) | ^12-14^ |
| 2020-03-26 | British Columbia placed restrictions on business operations, adopting approaches similar to Québec. | ^5^ |
| 2020-03-27 | Alberta ordered closure of all non-essential businesses. Gatherings of more than 15 people were prohibited in Alberta (CMOH order 07-2020). | ^15,16^ |
| 2020-03-30 | Ontario’s Chief Medical Officer of Health strongly recommended all people over 70 and those with compromised immune systems stop going out. Everyone else in the province should stay home as much as possible to limit the spread of COVID-19. | ^17^ |
| 2020-04-06 | Canada’s Chief Public Health Officer advised Canadians to wear “non-medical” masks to reduce the risk that asymptomatic people will spread SARS-CoV-2 | ^17^ |
| 2020-04-07 | Alberta imposed further restrictions on visitors at health care facilities in Alberta (CMOH order 09-2020 rescinding CMOH order 03-2020) | ^18^ |
| 2020-04-09 | Ontario broadened its criteria for testing people with symptoms of COVID-19 to include all hospital inpatients, long-term care residents, healthcare workers and caregivers, first responders, and residents of remote or Indigenous communities.  Ontario’s Medical Officer of Health also directed that all staff and essential visitors to long-term care homes must wear personal protective equipment for the entirety of their shift or visit, regardless of whether the home is experiencing an outbreak.  All residents of British Columbia returning home from abroad would have to submit detailed quarantine plans or be taken to a federal quarantine site. | ^17^ |
| 2020-04-15 | In Ontario, all symptomatic staff and residents of long-term care facilities would be screened, and staff are no longer allowed to work in more than one home. | ^17^ |
| 2020-04-28 | Alberta updated restrictions on visitors at long-term care and licensed supportive living facilities in Alberta (CMOH order 14-2020 rescinding CMOH order 19-2020) | ^19^ |
| 2020-05-01 | Alberta started slowly reopening provincial parks and golf courses (CMOH order 15-2020). | ^17,20^ |
| 2020-05-03 | Alberta resumed non-essential health services (e.g., elective surgeries and other non-urgent medical services) (CMOH order 16-2020). | ^21^ |
| 2020-05-05 | Alberta removed the prohibition from attending an outdoor shooting range (CMOH order 17-2020). | ^22^ |
| 2020-05-06 | British Columbia began to reopen its economy, easing lockdown restrictions on some summer sports activities, retail businesses and hair salons. | ^17^ |
| 2020-05-14 | Alberta allowed attendance to retail businesses, museums, hair salons, restaurants, daycares, out-of-school care and post-secondary institutions. However, schools and certain businesses/entities remain closed (including, personal service, wellness service, non-essential health service, recreational or entertainment business and festival or event business) (CMOH order 18-2020 and 19-2020). | ^23,24^ |
| 2020-05-15 | Alberta allowed attendance at gatherings of up to 50 people in a group in an outdoor location (CMOH order 20-2020). | ^25^ |
| 2020-05-19 | Ontario began to ease pandemic restrictions, gradually resuming scheduled surgeries and reopening some businesses and services, including retail stores with street entrances, construction, library pickups and some outdoor sporting competitions. Workplaces could also gradually reopen, but people should work from home as much as possible. The province also expanded eligibility for testing to include anyone with symptoms of COVID-19.  British Columbia began to ease pandemic restrictions, allowing various businesses, restaurants, and childcare to reopen as long as social distancing (2m distance) is maintained. Restaurants could have guests up to 6 people in a group. | ^26,27^ |
| 2020-05-20 | Ontario recommended that anyone over age two should wear a face covering when riding public transit unless they have trouble breathing or removing a mask. Provincial officials also said that transit agencies should erect plexiglass barriers on vehicles, make hand sanitizer available and clear high- touch areas. | ^17^ |
| 2020-05-21 | Ontario Premier promised to ramp up testing in the next three to four weeks, but the province was only completing about half its target of 16,000 tests per day. | ^17^ |
| 2020-05-27 | Ontario extended pandemic restrictions to June 9 that would have expired on May 29. Under the extended orders, playgrounds and pools would remain closed, bars and restaurants may only provide takeout and delivery services, and people may not gather in groups larger than five people. | ^17^ |
| 2020-05-27 | Alberta allowed preschool programs to operate (CMOH order 24-2020). | ^28^ |
| 2020-05-29 | Ontario started rolling out an expanded testing strategy for COVID-19, which included testing workers in key sectors and communities experiencing outbreaks, as well as asymptomatic individuals. Provincial labs hit testing targets of 16000 tests a day. | ^17^ |
| 2020-06-01 | The Canada federal government opened 38 national parks and 171 historic sites for hikes and other daytime activities while camping overnight was not yet permitted.  Ontario reopened provincial parks, drive-in movie theatres and batting cages.  British Columbia reopened schools on a part-time basis. | ^17^ |
| 2020-06-07 | Alberta began distributing 20 million non-medical masks at almost 600 drive-through locations across the province, including fast food restaurants. While mask use was not mandatory, Alberta’s chief medical officer recommended people wear a non-medical mask when physical distancing is not possible. Calgary and Edmonton would distribute an additional 500,000 masks through the transit system. | ^17^ |
| 2020-06-12 | 24 of Ontario’s 34 public health units started second phase of easing pandemic restrictions, and 10 other Greater Toronto and Hamilton Area and near the U.S. border would wait until new daily cases in those areas consistently decrease.  The second phase increased the limit on gatherings to 10 people and allowed selected personal care services, outdoor dining, and some outdoor recreational facilities to resume operations. | ^27^ |
| 2020-06-12 | Alberta began its Stage 2 relaunch. Alberta removed restrictions on public access to businesses, schools and places of worship as long as they implemented practices to minimize COVID-19 transmission risk, provide procedures for rapid response if a person develops symptoms and ensure that persons therein maintain high levels of sanitation and personal hygiene. Alberta CMOH order 25-2020, which rescinded previous CMOH orders: 01-2020, 02-2020, 07-2020, 15-2020, 16-2020, 17-2020, 18-2020, 19-2020 and 24-2020. | ^29^ |
| 2020-06-15 | Alberta lifted state of public health emergency. | ^30^ |
| 2020-06-18 | Ontario began allowing visitors to long-term care and retirement homes that do not have active cases of COVID-19. | ^27^ |
| 2020-06-19 | Ontario allowed the following public health unit regions to move into stage 2: Durham region health department, Haldimand-Norfolk health unit, Halton region health department, Hamilton public health services, Lambton health unit, Niagara region public health department, and York region public health services | ^27^ |
| 2020-06-24 | Ontario allowed Peel public health and Toronto public health units to move into stage 2.  British Columbia entered into phase 3 of reopening, which allowed overnight camping in parks. | ^27,31^ |
| 2020-06-25 | Ontario allowed the City of Windsor and all other parts of Essex County to enter stage 2, except the Municipality of Leamington and the Town of Kingsville | ^27^ |
| 2020-06-26 | Alberta required mandatory physical distancing of at least two meters from every other person who is not a member of the same household or cohort (CMOH order 26-2020). | ^32^ |
| 2020-07-01 | British Columbia allowed long-term care facility residents to have visit with one designated family member or friend. | ^31^ |
| 2020-07-07 | Ontario allowed the Municipality of Leamington and Town of Kingsville to enter stage 2. | ^27^ |
| 2020-07-17 | Ontario allowed the majority of their public health units to move into stage 3. | ^27^ |
| 2020-07-23 | Alberta permitted, with conditions, visitors to long-term care, licensed supportive living facility or any residential facility offering hospice services (CMOH order 29-2020 rescinding CMOH order 14-2020) | ^33^ |
| 2020-07-24 | Ontario allowed the following public health unit regions to move into stage 3: Durham region health department, Haldimand-Norfolk health unit, Halton region health department, Hamilton public health services, Lambton health unit, Niagara region public health department and York region public health services. | ^27^ |
| 2020-07-31 | Ontario allowed Peel public health and Toronto public health to move into stage 3. | ^27^ |
| 2020-08-12 | Ontario allowed Windsor-Essex County health unit to move into stage 3. | ^27^ |
| 2020-08-31 | Alberta required the use of non-medical mask for Grade 4 to 12 students, all staff and visitors in indoor spaces including on school buses and shared areas such as hallways (CMOH order 33-2020) | ^34^ |
| 2020-09-08 | Ontario paused any further loosening of public health measures or reopening of businesses, facilities or organizations. | ^35^ |
| 2020-09-22 | Indoor children’s play centers are allowed to open in Alberta. However, amusement park or nightclubs remain closed to the public (CMOH order 34-2020 amending CMOH order 25-2020). | ^36^ |
| 2020-10-26 | Alberta limited all private social gatherings in Edmonton and Calgary to a maximum of 15 people (CMOH order 35-2020). | ^37^ |
| 2020-11-06 | Alberta limited all private social gatherings to a maximum of 15 people in any area where the active case rate for the region is identified as 50 or greater (per 100,000 population); or the active cases for the region is identified as 10 or greater (CMOH order 36-2020 rescinding CMOH order 35-2020). | ^38^ |
| 2020-11-13 | Calgary and Edmonton Metropolitan regions in Alberta are prohibited from providing or hosting an indoor group fitness activity or sport activity, group performance activity (Nov 13 to Nov 27); all weddings or funeral ceremonies limited to max of 50 persons in specific locations; prohibited from selling or serving liquor after 10 pm and closed to public after 11pm (CMOH order 37-2020) | ^39^ |
| 2020-11-19 | British Columbia entered into provincewide restrictions and suspended all events and social gatherings on November 19, 2020 and the restrictions will be in effect until January 8, 2021 at midnight. | ^40^ |
| 2020-11-24 | Alberta prohibited private social gathering at an indoor public place except for funerals or weddings. Funerals or weddings were limited to 10 persons or less. Religious service was allowed if the number of persons attending the service was limited to 1/3 of the usual attendance of the place of worship and all persons therein must wear a face mask. Facemask are required at all times while attending an indoor public place in the metropolitan regions of Calgary and Edmonton (CMOH order 38-2020 rescinding CMOH order 36-2020 and part 3 of CMOH order 37-2020). | ^41^ |
| 2020-11-27 | Alberta updated their order with regard to what businesses or entities that must be closed to the public, that must limit capacity and that must schedule an appointment in order to provide services (CMOH order 39-2020 rescinding part 1, 2 and 4 of CMOH order 37-2020). | ^42^ |
| 2020-11-28 | Alberta updated their requirement regarding places of worship. Religious service is limited to 1/3 of the total operational occupant load and all worshippers should wear a face mask (CMOH order 40-2020 amending CMOH order 38-2020 and 39-2020). | ^43^ |
| 2020-12-08 | Alberta entered into provincewide restrictions and suspended all indoor and outdoor social gatherings on December 8, 2020 and the restrictions will be in effect at least until January 12, 2021. Masks were made mandatory provincewide (CMOH order 41-2020 amending CMOH order 38-2020). | ^44,45^ |
| 2020-12-13 | Effective on December 13, 2020, Alberta placed further restrictions on private social gatherings. However, funerals and weddings of 10 persons or less were allowed to occur at an indoor or outdoor public or private place. Religious service is limited to 15% of the total operational occupant; drive-in religious service was allowed as long as participants remained in their vehicles (CMOH order 42-2020 rescinding 38-2020 and 39-2020). | ^46^ |
| 2020-12-23 | Alberta allowed for counselling services and a visit at home between a person who is at the end of their life (last 4 to 6 weeks, as determined by that person’s primary health care provider) and a family member, friend, faith leader or other person as long as no more than 3 visitors enter the private residence of the dying person at one time (CMOH order 44-2020 amending CMOH order 42-2020 and rescinding CMOH order 43-2020). | ^47^ |
| 2020-12-26 | Ontario entered into provincewide shutdown on December 26, 2020 at 12:01am and the restrictions will be in effect for all of Ontario from December 26, 2020 to January 9, 2021. For 27 public health unit regions in Southern Ontario, those restrictions will remain in effect from January 9 to January 23, 2021. | ^48^ |

**Table S2.** The assumed date of infection of first cases and the cumulative number of cases as of December 1, 2020 by provincial and subprovincial jurisdiction.

|  | The approximate date of infection of the first case in the data set* | Cumulative number of cases as of December 1, 2020** |
| --- | --- | --- |
| **Alberta** | **2020-02-26** | **61169** |
| Calgary Zone, AB | 2020-02-26 | 24983 |
| Central Zone, AB | 2020-03-02 | 3028 |
| Edmonton Zone, AB | 2020-02-29 | 24895 |
| North Zone, AB | 2020-03-03 | 4045 |
| South Zone, AB | 2020-03-07 | 3987 |
| Location unknown | Not applied | 231 |
| **British Columbia** | **2020-01-24** | **34699** |
| Fraser Health Authority, BC | 2020-02-11 | 22007 |
| Interior Health Authority, BC | 2020-02-02 | 1899 |
| Northern Health Authority, BC | 2020-02-29 | 905 |
| Vancouver Coastal Health Authority, BC | 2020-01-24 | 9135 |
| Vancouver Island Health Authority, BC | 2020-03-01 | 659 |
| **Ontario** | **2019-12-19** | **121745** |
| Algoma Public Health Unit, ON | 2020-03-02 | 61 |
| Brant County Health Unit, ON | 2020-02-29 | 613 |
| Chatham-Kent Health Unit, ON | 2020-03-01 | 515 |
| Durham Region Health Department, ON | 2020-02-18 | 4694 |
| Eastern Ontario Health Unit, ON | 2020-01-10 | 872 |
| Grey Bruce Health Unit, ON | 2020-02-29 | 329 |
| Haldimand-Norfolk Health Unit, ON | 2019-12-26 | 645 |
| Haliburton, Kawartha, Pine Ridge District Health Unit, ON | 2020-02-25 | 328 |
| Halton Region Health Department, ON | 2020-02-25 | 3643 |
| Hamilton Public Health Services, ON | 2020-02-14 | 3351 |
| Hastings and Prince Edward Counties Health Unit, ON | 2020-01-26 | 154 |
| Huron Perth District Health Unit, ON | 2020-03-01 | 359 |
| Kingston, Frontenac and Lennox & Addington Public Health, ON | 2020-02-28 | 265 |
| Lambton Public Health, ON | 2020-02-29 | 417 |
| Leeds, Grenville and Lanark District Health Unit, ON | 2020-02-24 | 498 |
| Middlesex-London Health Unit, ON | 2020-01-18 | 1699 |
| Niagara Region Public Health Department, ON | 2020-02-29 | 2203 |
| North Bay Parry Sound District Health Unit, ON | 2020-03-03 | 79 |
| Northwestern Health Unit, ON | 2020-03-06 | 121 |
| Ottawa Public Health, ON | 2020-02-04 | 8606 |
| Peel Public Health, ON | 2020-01-25 | 26854 |
| Peterborough Public Health, ON | 2020-02-21 | 216 |
| Porcupine Health Unit, ON | 2020-02-28 | 107 |
| Renfrew County and District Health Unit, ON | 2020-03-04 | 133 |
| Simcoe Muskoka District Health Unit, ON | 2020-02-26 | 2199 |
| Southwestern Public Health, ON | 2020-02-24 | 579 |
| Sudbury & District Health Unit, ON | 2020-02-26 | 231 |
| Thunder Bay District Health Unit, ON | 2020-02-23 | 331 |
| Timiskaming Health Unit, ON | 2020-03-15 | 18 |
| Toronto Public Health, ON | 2019-12-23 | 41261 |
| Region of Waterloo, Public Health, ON | 2019-12-26 | 3804 |
| Wellington-Dufferin-Guelph Public Health, ON | 2020-02-14 | 1435 |
| Windsor-Essex County Health Unit, ON | 2020-02-17 | 3795 |
| York Region Public Health Services, ON | 2019-12-19 | 11330 |

*The dates for Alberta and British Columbia are dates of report minus 9 days. The dates of Ontario are accurate episode dates minus 6 days. ** Cumulative number of cases as of December 1, 2020. Cases with date of report of December 2, 2020 (British Columbia) or accurate episode date of December 2, 2020 (Ontario) were excluded.

**Table S3.** Cross-tabulation of Ontario cases* by their case acquisition information and their association with COVID-19 outbreaks in congregate settings.

|  | Case count (row %) associated with COVID-19 outbreaks in congregate settings** | |  |
| --- | --- | --- | --- |
| Case Acquisition Info | Yes | No | Row sum (column %) |
| Close contact | 3244 (6.53) | 46458 (93.47) | 49702 (40.82) |
| Missing Information | 65 (1.78) | 3596 (98.22) | 3661 (3.01) |
| No known epi link | 1730 (4.45) | 37173 (95.55) | 38903 (31.95) |
| Unspecified epi link | 6 (2.75) | 212 (97.25) | 218 (0.18) |
| Outbreak | 25347 (97.28) | 709 (2.72) | 26056 (21.40) |
| Travel | 46 (1.44) | 3159 (98.56) | 3205 (2.63) |
| TOTAL | 30438 (25.00) | 91307 (75.00) | 121745 (100) |

*In our dataset, we included cases with accurate episode dates up to December 1, 2020. **This is the Variable: “Outbreak_Related” in the dataset.

**Table S4.** Cumulative case count by congregate or non-congregate setting in Ontario and health authorities with a cumulative number of 2000 cases or more, as of December 1, 2020.

| Jurisdiction | Congregate setting, n(%) | Non-congregate setting, n(%) | Total |
| --- | --- | --- | --- |
| Ontario | 30438 (25.00) | 91307 (75.00) | 121745 |
| Toronto | 10477 (25.39) | 30784 (74.61) | 41261 |
| Peel | 3566 (13.28) | 23288 (86.72) | 26854 |
| York | 2401 (21.19) | 8929 (78.81) | 11330 |
| Ottawa | 3856 (44.81) | 4750 (55.19) | 8606 |
| Durham | 1462 (31.15) | 3232 (68.85) | 4694 |
| Waterloo | 989 (26.00) | 2815 (74.00) | 3804 |
| Windsor | 1685 (44.40) | 2110 (55.60) | 3795 |
| Halton | 703 (19.30) | 2940 (80.70) | 3643 |
| Hamilton | 865 (25.81) | 2486 (74.19) | 3351 |
| Niagara | 949 (43.08) | 1254 (56.92) | 2203 |
| Simcoe | 478 (21.74) | 1721 (78.26) | 2199 |

**Table S5.** Cumulative case count per 100,000 and incidence rate ratio between Alberta, British Columbia and Ontario by sex (or gender) and age group, as of December 1, 2020.

|  | **Cumulative case count per 100,000** | | | **Incidence Rate Ratio** | | |
| --- | --- | --- | --- | --- | --- | --- |
| **Female** | **AB** | **BC** | **ON** | **AB** | **BC** | **ON** |
| <20 | 1458.8 | 436.4 | 456.8 | 3.3 | Ref | 1.0 |
| 20-29 | 2015.6 | 1142.7 | 1195.8 | 1.8 | Ref | 1.0 |
| 30-39 | 1669.6 | 884.5 | 924.4 | 1.9 | Ref | 1.0 |
| 40-49 | 1649.9 | 794.5 | 942.6 | 2.1 | Ref | 1.2 |
| 50-59 | 1275.8 | 599.4 | 893.8 | 2.1 | Ref | 1.5 |
| 60-69 | 904.0 | 381.1 | 615.6 | 2.4 | Ref | 1.6 |
| 70-79 | 783.9 | 362.6 | 535.0 | 2.2 | Ref | 1.5 |
| 80+ | 1537.8 | 738.9 | 1738.6 | 2.1 | Ref | 2.4 |
| All age groups | 1390.6 | 662.4 | 836.8 | 2.1 | Ref | 1.3 |
| **Male** | **AB** | **BC** | **ON** | **AB** | **BC** | **ON** |
| <20 | 1461.3 | 446.9 | 473.7 | 3.3 | Ref | 1.1 |
| 20-29 | 1946.0 | 1169.2 | 1237.4 | 1.7 | Ref | 1.1 |
| 30-39 | 1706.0 | 930.7 | 983.5 | 1.8 | Ref | 1.1 |
| 40-49 | 1643.2 | 800.3 | 885.1 | 2.1 | Ref | 1.1 |
| 50-59 | 1274.9 | 643.3 | 817.5 | 2.0 | Ref | 1.3 |
| 60-69 | 982.4 | 450.4 | 710.2 | 2.2 | Ref | 1.6 |
| 70-79 | 875.2 | 422.6 | 613.4 | 2.1 | Ref | 1.5 |
| 80+ | 1464.3 | 706.7 | 1268.3 | 2.1 | Ref | 1.8 |
| All age groups | 1404.0 | 700.3 | 824.6 | 2.0 | Ref | 1.2 |
| **Total** | **AB** | **BC** | **ON** | **AB** | **BC** | **ON** |
| <20 | 1462.4 | 442.2 | 468.8 | 3.3 | Ref | 1.1 |
| 20-29 | 1982.0 | 1158.2 | 1225.0 | 1.7 | Ref | 1.1 |
| 30-39 | 1690.4 | 908.7 | 958.9 | 1.9 | Ref | 1.1 |
| 40-49 | 1648.2 | 797.9 | 920.7 | 2.1 | Ref | 1.2 |
| 50-59 | 1276.6 | 621.5 | 859.8 | 2.1 | Ref | 1.4 |
| 60-69 | 943.7 | 415.5 | 663.7 | 2.3 | Ref | 1.6 |
| 70-79 | 827.7 | 392.2 | 574.3 | 2.1 | Ref | 1.5 |
| 80+ | 1509.4 | 725.2 | 1565.0 | 2.1 | Ref | 2.2 |
| All age groups | 1399.3 | 684.2 | 835.8 | 2.0 | Ref | 1.2 |

AB: Alberta; BC: British Columbia; ON: Ontario. Ref: Reference group.

**Table S6.** Cumulative case count and cumulative case count per 100,000 population by age and gender in Alberta (N=61,169), as of the date of report of December 1, 2020.

|  | Cumulative case count | | |  |  | Population (2019) | |  | Cumulative case count per 100,000 | | |  |
| --- | --- | --- | --- | --- | --- | --- | --- | --- | --- | --- | --- | --- |
| Age group (year) | F | M | Unk | % female by age group | Total by age group (%) | F | M | Total | F | M | Total | |
| 0-4 | 1051 | 1072 | 6 | 49.4 | 2129 | 134487 | 139868 | 274355 | 781.5 | 766.4 | 776.0 | |
| *<1* | *167* | *175* | *6* | *48.0* | *348* | No data | No data | No data | n/a | n/a | n/a | |
| *1-4* | *884* | *897* | *0* | *49.6* | *1781* | No data | No data | No data | n/a | n/a | n/a | |
| 5-9 | 1182 | 1298 | 2 | 47.6 | 2482 | 135170 | 141947 | 277117 | 874.4 | 914.4 | 895.7 | |
| 10-19 | 3617 | 3751 | 11 | 49.0 | 7379 | 131345 | 137050 | 268395 | 2753.8 | 2737.0 | 2749.3 | |
| 20-29 | 5842 | 6039 | 14 | 49.1 | 11895 | 289835 | 310327 | 600162 | 2015.6 | 1946.0 | 1982.0 | |
| 30-39 | 5839 | 6120 | 17 | 48.8 | 11976 | 349731 | 358741 | 708472 | 1669.6 | 1706.0 | 1690.4 | |
| 40-49 | 4842 | 4968 | 10 | 49.3 | 9820 | 293475 | 302344 | 595819 | 1649.9 | 1643.2 | 1648.2 | |
| 50-59 | 3503 | 3560 | 7 | 49.5 | 7070 | 274579 | 279245 | 553824 | 1275.8 | 1274.9 | 1276.6 | |
| 60-69 | 2073 | 2232 | 3 | 48.1 | 4308 | 229311 | 227188 | 456499 | 904.0 | 982.4 | 943.7 | |
| 70-79 | 1012 | 1023 | 1 | 49.7 | 2036 | 129090 | 116890 | 245980 | 783.9 | 875.2 | 827.7 | |
| 80+ | 1239 | 809 | 2 | 60.4 | 2050 | 80569 | 55247 | 135816 | 1537.8 | 1464.3 | 1509.4 | |
| Unknown | 2 | 7 | 15 | 8.3 | 24 | n/a | n/a | n/a | n/a | n/a | n/a | |
| All age groups | 30202 | 30879 | 88 | 49.4 | 61169 | 2171882 | 2199434 | 4371316 | 1390.6 | 1404.0 | 1399.3 | |

F: female; M: male; n/a: not applied.

**Table S7.** Cumulative case count and cumulative case count per 100,000 population by age and sex in British Columbia (N=34,699), as of the date of report of December 1, 2020.

|  | Cumulative case count | | |  |  | Population (2019) | |  | Cumulative case count per 100,000 | |  |
| --- | --- | --- | --- | --- | --- | --- | --- | --- | --- | --- | --- |
| Age group (year) | F | M | Unk | % female by age group | Total by age group (%) | F | M | Total | F | M | Total |
| <10 | 651 | 711 | 2 | 47.7 | 1364 | 227170 | 241751 | 468921 | 286.6 | 294.1 | 290.9 |
| 10-19 | 1456 | 1559 | 2 | 48.3 | 3017 | 255594 | 266185 | 521779 | 569.7 | 585.7 | 578.2 |
| 20-29 | 3796 | 4190 | 12 | 47.5 | 7998 | 332196 | 358356 | 690552 | 1142.7 | 1169.2 | 1158.2 |
| 30-39 | 3155 | 3361 | 7 | 48.4 | 6523 | 356694 | 361142 | 717836 | 884.5 | 930.6 | 908.7 |
| 40-49 | 2628 | 2526 | 4 | 50.9 | 5158 | 330762 | 315651 | 646413 | 794.5 | 800.3 | 797.9 |
| 50-59 | 2224 | 2269 | 5 | 49.4 | 4498 | 371018 | 352695 | 723713 | 599.4 | 643.3 | 621.5 |
| 60-69 | 1299 | 1435 | 6 | 47.4 | 2740 | 340886 | 318620 | 659506 | 381.1 | 450.4 | 415.5 |
| 70-79 | 778 | 840 | 3 | 48.0 | 1621 | 214540 | 198754 | 413294 | 362.6 | 422.6 | 392.2 |
| 80-89 | 572 | 505 | 0 | 53.1 | 1077 | 100562 | 82112 | 182674 | 568.8 | 615.0 | 589.6 |
| 90+ | 401 | 185 | 0 | 68.4 | 586 | 31125 | 15523 | 46648 | 1288.4 | 1191.8 | 1256.2 |
| Unknown | 0 | 1 | 116 | 0 | 117 | n/a | n/a |  | n/a | n/a |  |
| All age groups | 16960 | 17582 | 157 | 48.9 | 34699 | 2560547 | 2510789 | 5071336 | 662.4 | 700.3 | 684.2 |

F: female; M: male; n/a: not applied.

**Table S8.** Cumulative case count and cumulative case count per 100,000 population by age and gender in Ontario (N=121,745), as of the accurate episode date of December 1, 2020.

|  | Cumulative case count | | | |  |  | Population (2019) | |  | Cumulative case count per 100,000 | |  |
| --- | --- | --- | --- | --- | --- | --- | --- | --- | --- | --- | --- | --- |
| Age group (year) | F | M | Gender Diverse | Un-specified | % female by age group | Total by age group (%) | F | M | Total | F | M | Total |
| <20 | 7012 | 7610 | 4 | 103 | 47.6 | 14729 | 1535170 | 1606523 | 3141693 | 456.8 | 473.7 | 468.8 |
| 20-29 | 11996 | 13396 | 3 | 155 | 47.0 | 25550 | 1003139 | 1082560 | 2085699 | 1195.8 | 1237.4 | 1225.0 |
| 30-39 | 9147 | 9700 | 2 | 97 | 48.3 | 18946 | 989503 | 986267 | 1975770 | 924.4 | 983.5 | 958.9 |
| 40-49 | 8970 | 8006 | 4 | 110 | 52.5 | 17090 | 951581 | 904542 | 1856123 | 942.6 | 885.1 | 920.7 |
| 50-59 | 9278 | 8351 | 2 | 77 | 52.4 | 17708 | 1037994 | 1021545 | 2059539 | 893.8 | 817.5 | 859.8 |
| 60-69 | 5465 | 5885 | 2 | 40 | 48.0 | 11392 | 887750 | 828658 | 1716408 | 615.6 | 710.2 | 663.7 |
| 70-79 | 3108 | 3115 | 1 | 29 | 49.7 | 6253 | 580926 | 507818 | 1088744 | 535.0 | 613.4 | 574.3 |
| 80-89 | 3794 | 2248 | 0 | 51 | 62.3 | 6093 | 295818 | 216615 | 512433 | 1282.5 | 1037.8 | 1189.0 |
| 90+ | 2905 | 1014 | 0 | 44 | 73.3 | 3963 | 89561 | 40577 | 130138 | 3245.8 | 2499.0 | 3045.2 |
| Unk | 3 | 3 | 0 | 2 | 37.5 | 8 | n/a | n/a | n/a | n/a | n/a | n/a |
| n/a | 6 | 3 | 0 | 4 | 46.2 | 13 | n/a | n/a | n/a | n/a | n/a | n/a |
| All age groups | 61684 | 59331 | 18 | 712 | 50.7 | 121745 | 7371442 | 7195105 | 14566547 | 836.8 | 824.6 | 835.8 |

F: female; M: male; n/a: not applied; Unk: Unknown.
